# Molecular-Level Insights into the Spatial Distribution, Transformation, and Potential Sources of Dissolved Organic Nitrogen in the Beidagang Reservoir

**DOI:** 10.3390/molecules31203774

**Published:** 2026-10-21

**Authors:** Lin Diao, Xiaomin Wang, Sihui Su, Xiaodong Zhang, Cuixia Zhang, Mengdi Li, Haiming Li

**Affiliations:** 1 College of Marine and Environmental Sciences, Tianjin University of Science and Technology, Tianjin 300457, China; 2 Laboratory of Coastal Groundwater Utilization and Protection, Tianjin University of Science and Technology, Tianjin 300457, China

**Keywords:** dissolved organic nitrogen, Fourier transform ion cyclotron resonance mass spectrometry, molecular composition, Beidagang Reservoir

## Abstract

Dissolved organic nitrogen (DON) is an important component of the aquatic nitrogen pool, which is composed of countless molecular individuals with different ecological functions. Yet, its sources and molecular composition remain poorly understood in reservoir ecosystems. In this study, water samples were collected from different regions of the Beidagang Reservoir. Hydrochemical measurements and Fourier transform ion cyclotron resonance mass spectrometry (FT-ICR MS) were used to characterize the molecular composition, spatial distribution, and potential sources of DON. The results demonstrated that DON constituted a substantial fraction of total dissolved nitrogen and exhibited pronounced spatial variability in both concentration and molecular composition. CHO and CHON formulas were abundant across the reservoir, with carboxylic-rich alicyclic molecules (CRAM-like) representing the dominant molecular class. A total of 16,338 CHON formulas were identified, of which only 407 were shared by all samples, indicating pronounced spatial variation in DON composition. Paired mass distance (PMD) analysis further showed that approximately 60% of the DON formulas could be classified as potential precursors or products. Amine reactions accounted for approximately 40% of all putative transformation pairs, suggesting that transformations involving nitrogen functional groups may represent an important pathway during DON transport. Upstream DON was likely influenced by the combined effects of surface runoff, atmospheric deposition, and internal reservoir processes, whereas downstream DON appeared to be more strongly affected by anthropogenic activities and seawater-related sources. These findings revealed the molecular diversity and potential transformation pathways of DON in the Beidagang Reservoir and provided molecular-level evidence for understanding nitrogen cycling in reservoirs exposed to multiple environmental inputs, which helped predict the response of nitrogen cycling to global climate change.

## 1. Introduction

Nitrogen is an essential nutrient in aquatic ecosystems and occurs in multiple chemical forms that are directly affected by biodiversity and water quality [1]. Dissolved organic nitrogen (DON) is a major component of dissolved nitrogen and dissolved organic matter, encompassing diverse compounds such as urea, amino acids, proteins, nucleic acids, amino sugars, and humic substances [2]. Some DON compounds are readily utilized by microorganisms and actively participate in aquatic nitrogen cycling, whereas others are more persistent and can be transported and continuously transformed within aquatic ecosystems [2,3]. Organic nitrogen compounds can also serve as important precursors of nitrogenous disinfection by-products in drinking-water treatment processes [4]. Therefore, characterizing the molecular composition and transformation of DON in reservoirs is essential for evaluating its ecological effects and potential implications for drinking-water safety.

The molecular composition of aquatic DON is jointly controlled by external inputs and internal biogeochemical processes. Surface runoff and anthropogenic activities introduce diverse organic nitrogen compounds, while microbial metabolism, photochemical reactions, and adsorption–desorption processes further modify their molecular characteristics [5,6,7]. Studies of the Rappbode and Muldenberg drinking-water reservoirs have used FT-ICR MS and complementary field and experimental evidence to investigate DOM processing [8,9]. These studies illustrate how molecular composition can be linked to photochemical and microbial processes. Bulk parameters, such as DON concentration, provide useful information on overall abundance but offer limited insight into molecular-level composition and potential transformation pathways. Fourier transform ion cyclotron resonance mass spectrometry (FT-ICR MS), with its ultrahigh mass resolution and accuracy, enables the assignment of nitrogen-containing molecular formulas and characterization of DON composition. It has been applied to investigate DON cycling in coastal seas, changes during river-to-sea transport, and compositional changes during sludge reject water treatment [3,7,10]. In addition, paired mass distance (PMD) analysis based on FT-ICR MS data can be used to infer potential transformation relationships among molecular formulas, thereby providing clues to the migration and transformation of DON within reservoir systems [11].

Reservoir operation can alter water residence time, hydrodynamic conditions, and river connectivity, thereby influencing the transport and transformation of dissolved organic matter [5,12]. The Beidagang Reservoir is an important water-regulation facility in Tianjin. The hydrochemical characteristics of the reservoir exhibited water salinization and spatial variability [13]. The molecular composition of DON in the reservoir may be affected by the surrounding environment, anthropogenic activities, and supplementary water sources. However, current understanding of nitrogen cycling in the Beidagang Reservoir is largely based on bulk indicators, including total nitrogen, inorganic nitrogen, and DON concentrations. Molecular-level information on the composition, spatial distribution, and potential transformation of reservoir DON remains limited.

For the current study, water samples were collected from different regions of the Beidagang Reservoir and analyzed by FT-ICR MS to characterize the molecular composition and spatial distribution of DON. Spatial variations in DON molecular diversity were assessed. And the potential influences of external inputs and internal biogeochemical processes on the molecular composition, migration, and transformation of DON were explored.

## 2. Results

### 2.1. Physicochemical Properties of Samples

Concentrations of DOC and nitrogen indices are presented in Table S1 and Figure S2a. Although ZLZ showed the lowest concentration of DON (2.24 mg L^−1^) among all samples, representing only 41.40% of TDN, DON still remains an important part of TDN in the Beidagang Reservoir. The concentration of TDN showed a wider range (4.83–23.80 mg L^−1^) than DOC (9.32–15.63 mg L^−1^), whereas their mean concentrations (11.60 mg L^−1^ and 11.02 mg L^−1^) were comparable. TDN concentrations were particularly high at NAAN and DG, reaching 21.75 and 23.80 mg L^−1^, respectively. DON accounted for more than 85% of TDN at both sites and at SHTJ, indicating that organic nitrogen dominated the dissolved nitrogen pool at these sites. Ammonium concentrations were higher than nitrate concentrations in all water samples, indicating that dissolved inorganic nitrogen (DIN) in the Beidagang Reservoir was dominated by ammonium.

Concentrations of inorganic ions are presented in Table S3 and Figure S2b. The ionic composition of water samples in the Beidagang Reservoir exhibited distinct spatial differences. The total inorganic ion concentration ranged from 22.18 to 72.19 meq L^−1^. Overall, the northern sites of the reservoir (NAAN, SHTJ, SHXS and PXZ) had higher ion concentrations than the southern sites (ZLZ, MJ, DG, LGZ, and SJZ). Within the southern part of the reservoir, total ion concentrations generally showed an increasing trend from west to east. Among cations, Na^+^ had the highest mean concentration (9.69 meq L^−1^), exceeding those of Mg^2+^ and Ca^2+^. The highest Na^+^ concentration was observed at SHTJ (22.56 meq L^−1^). Among anions, Cl^−^ had the highest mean concentration (12.84 meq L^−1^), exceeding those of SO_4_^2−^ and HCO_3_^−^. The two highest Cl^−^ concentrations were observed at SHTJ and PXZ, reaching 31.17 and 24.92 meq L^−1^. In general, Na^+^ and Cl^−^ were the dominant ions in most samples. However, a few sites showed different ionic patterns. For example, SO_4_^2−^ accounted for the largest proportion of anions at NAAN (42.1%), whereas HCO_3_^−^ was the predominant anion for MJ (55.79%).

### 2.2. Overall Molecular Characteristics

The reconstructed mass spectra of assigned formulas across all samples are presented in Figure S3. Molecular formulas were classified into four elemental groups (CHO, CHON, CHOS, and CHONS). Signal intensities were normalized separately for each sample, with the highest peak set to 100%. In most samples, peaks with high relative signal intensities were mainly distributed over 250–500 Da. At SHXS, peaks assigned to CHO formulas were mainly distributed over 400–700 Da. In general, the relative intensities were comparatively lower at PXZ, ZLZ, DG, LGZ, and SJZ. Reservoir water samples exhibited both common and sample-specific chemodiversity patterns in Table S4 and Figure S4. The number of CHO and CHON formulas dominated the assigned formulas, accounting for 63–71% of the total number. MJ and LGZ exhibited relatively higher contributions of CHON formulas, whereas PXZ presented a relatively higher contribution of CHOS and CHONS formulas.

**Figure 1:**
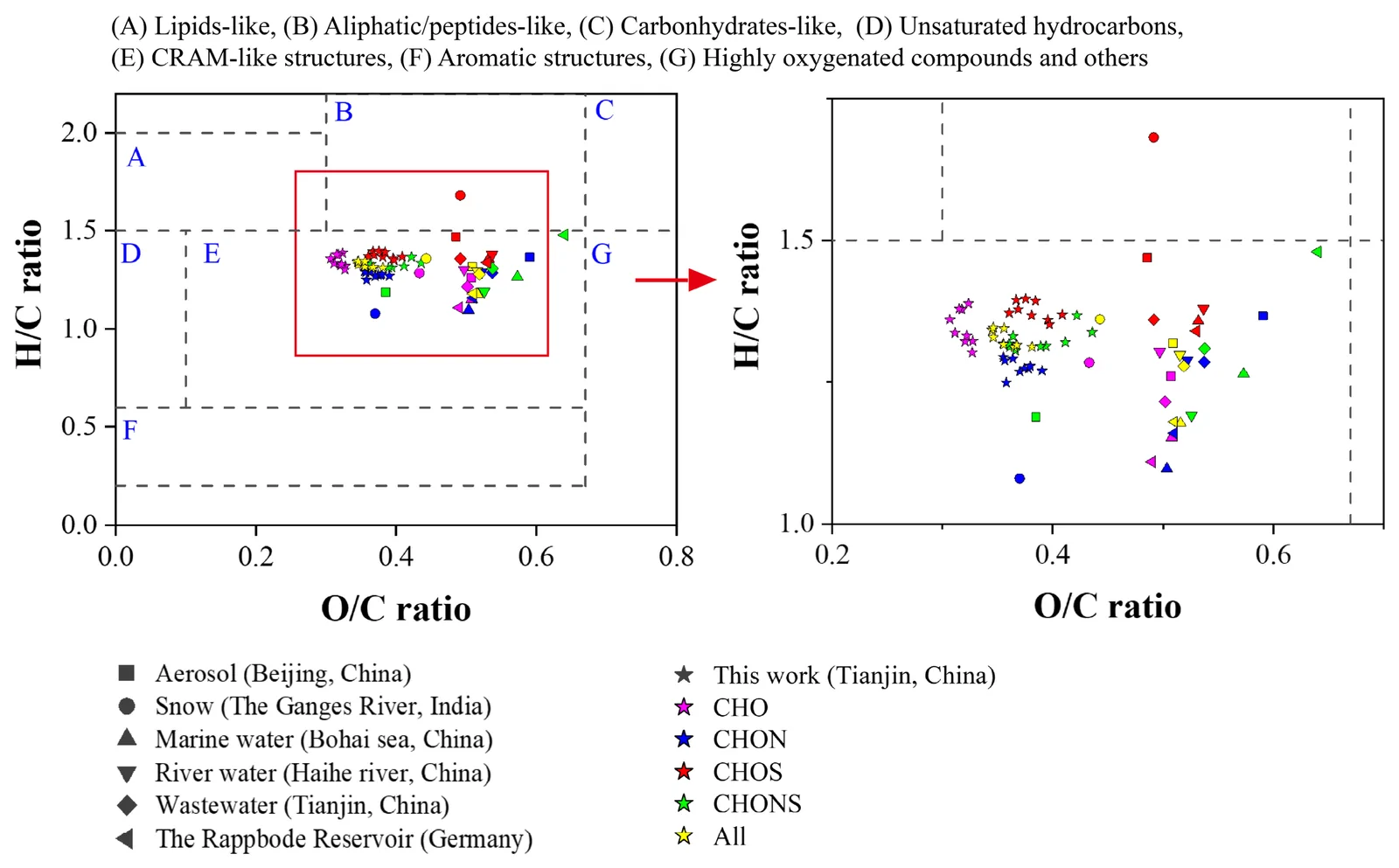
Comparison of average O/C and average H/C ratios for Beidagang Reservoir samples and published reference datasets, including aerosols, snow, marine water, river water, wastewater, and Rappbode Reservoir water. [9,14,15,16,17]. Values from this study are arithmetic means across assigned molecular formulas.

The arithmetic mean O/C and H/C ratios of all samples and their distributions in the VK diagrams are shown in Figure 1 and Table S5. The mean O/C ratios (0.35–0.38) and H/C ratios (1.31–1.35) exhibited more differences among compound classes than among sampling sites. CHO compounds had relatively low mean O/C ratios, whereas CHOS and CHONS compounds showed higher mean O/C ratios, with a maximum of 0.44 at PXZ. Their mean nominal oxidation states of carbon (NOSC) were also higher, ranging from −0.53 to −0.41 for CHOS and from −0.20 to 0.06 for CHONS, compared with −0.75 to −0.65 for CHO (Table S4). Thus, sulfur-containing formulas showed both higher oxygen content relative to carbon and higher mean NOSC than CHO formulas. The mean H/C ratios of CHON compounds were lower (1.25–1.29), while those of CHOS were higher (1.35–1.40). This indicates that CHON compounds are more unsaturated than CHOS molecules. Compared with other sample types reported in previous studies [9,14,15,16,17], reservoir water samples showed lower O/C ratios within the same compound classes. Their H/C ratios (1.30–1.40) were comparable to the values reported for aerosols, snow and river water but partly higher than those reported for marine water (1.10–1.36) and the Rappbode Reservoir (1.18–1.48). Overall, the Beidagang Reservoir is characterized by relatively low oxidation and high saturation. The factors contributing to this variability are explored in subsequent content. According to the VK diagram classification proposed by Bianco [18], the mean O/C and H/C ratios of the samples were predominantly located in region E (CRAM-like structures).

**Figure 2:**
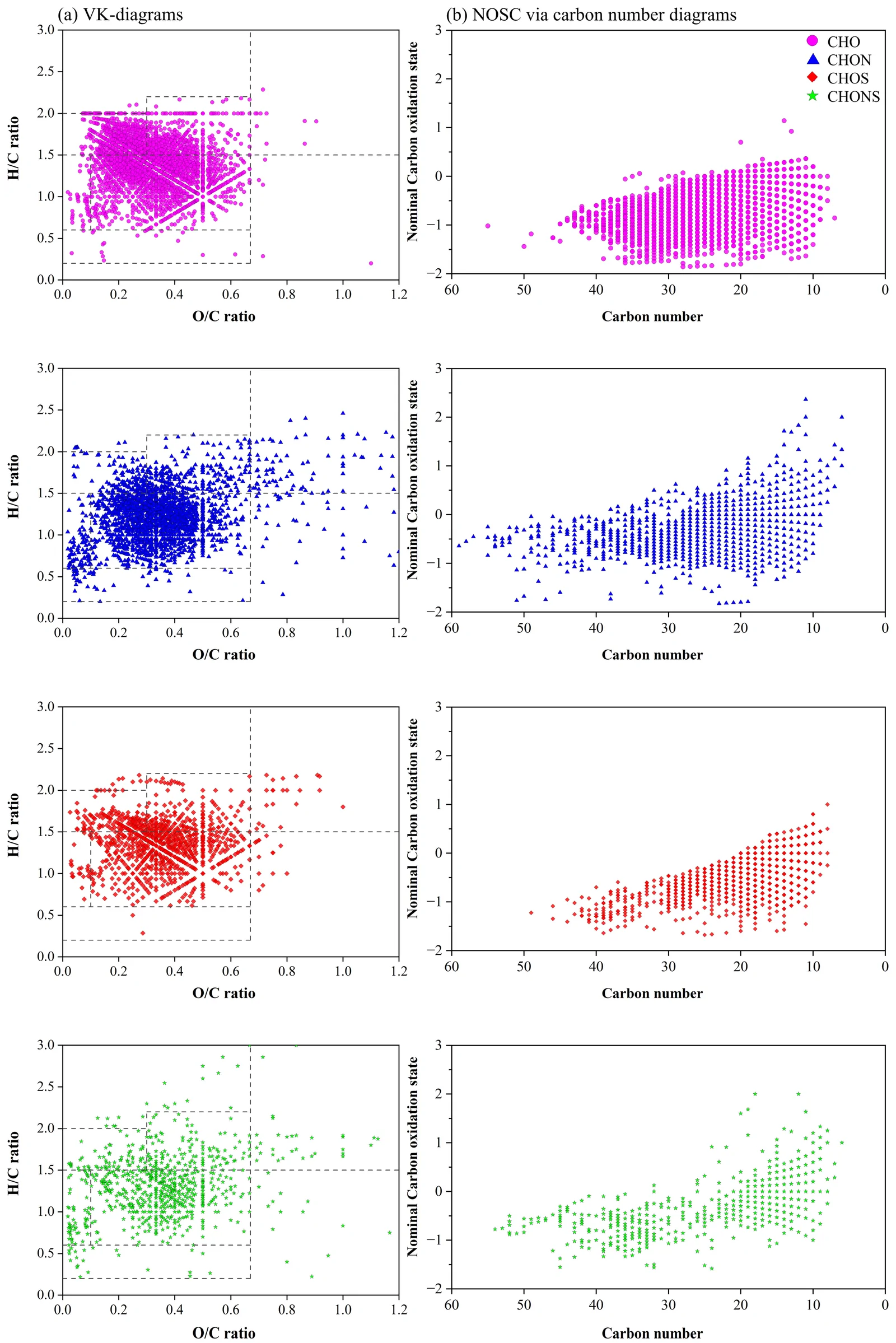
Van Krevelen diagrams (**a**) and nominal carbon oxidation state (NOSC) via carbon number (**b**) of assigned formulas in NAAN. CHO, CHON, CHOS, and CHONS formulas are shown separately from top to bottom. The dashed lines show the class identification of VK diagrams [18]. Corresponding diagrams for the remaining eight samples are provided in the Supporting Information.

The distributions of molecular formulas in the VK diagrams and nominal oxidation state of carbon (NOSC) via carbon number for all samples are shown in Figure 2 and Figures S7–S15, Tables S4 and S6. Region E dominated across all samples, accounting for approximately 60–65% of the assigned formulas. The proportion of region E was highest in DG and ZLZ (~64.7%) and lowest in LGZ and SJZ (~60%). Regions A (lipids-like) and B (aliphatic-like and peptide-like) followed, accounting for about 8–13% and 12–14% of the formulas. In contrast, region G (highly oxygenated compounds and others) represented a minor fraction, generally around 2%. Among the northern reservoir sites, the proportion of region C (carbohydrate-like) increased from 2.17% in NAAN to 5.57% in PXZ from west to east. A similar trend was also observed for region A across the southern reservoir sites. Most formulas were distributed within an intermediate carbon number range (C_10_-C_30_), with NOSC values primarily between −2 and 0. Overall, the NOSC distribution was broader in the region with lower carbon numbers, with a greater extension toward higher NOSC values. Although the distribution ranges were comparable among all samples, upstream samples such as NAAN had relatively more formulas in the higher carbon number range, whereas downstream samples such as PXZ contained relatively more formulas in the low-NOSC region. Mean NOSC followed the order CHONS > CHON > CHOS > CHO in all samples (Table S4).

### 2.3. Characterization of CHON Formulas Shared by All Samples

**Figure 3:**
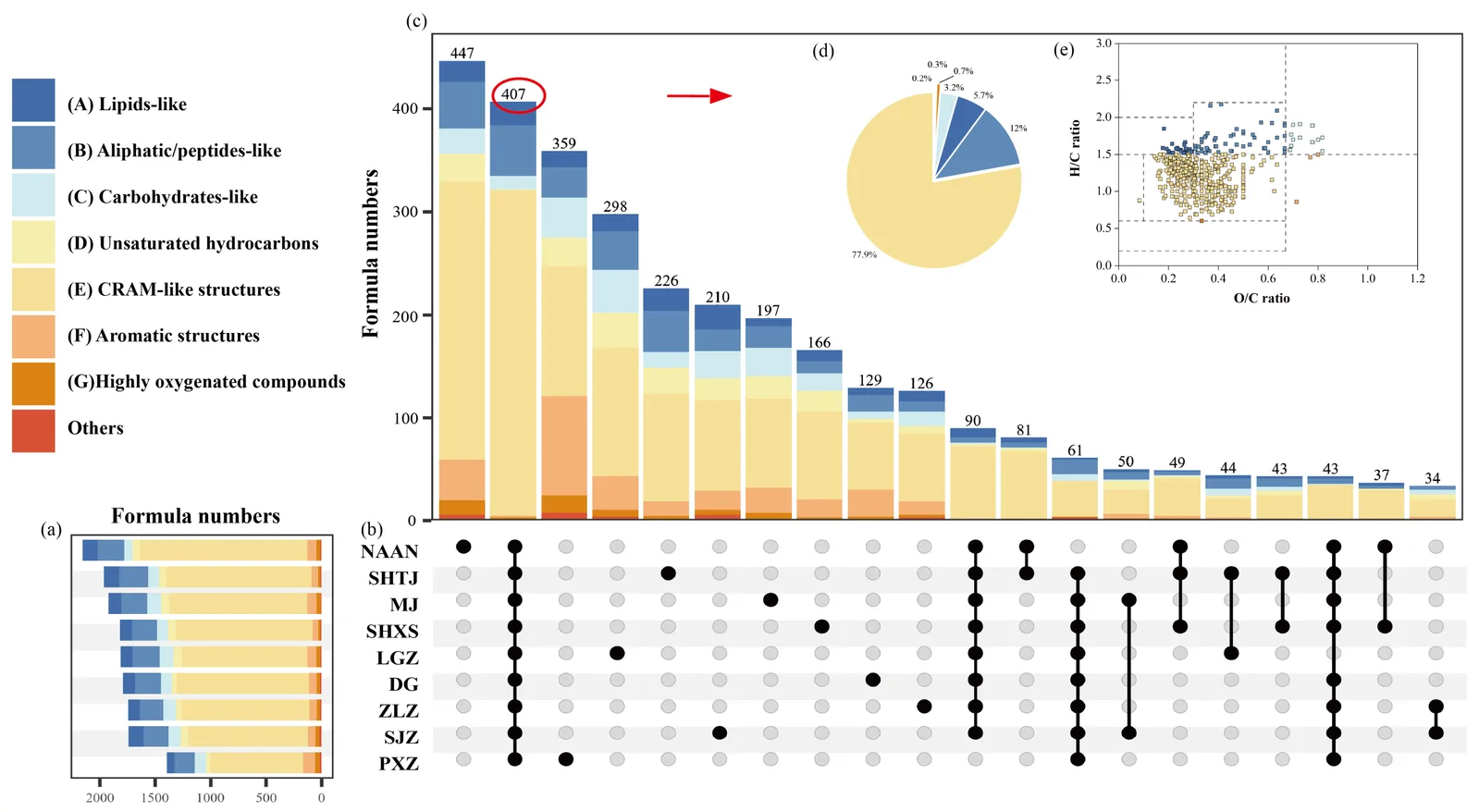
Differences in CHON formulas among the nine samples. (**a**) Numbers of CHON formulas and the proportion of each class in each sample [18]. (**b**) CHON formulas are unique (black spot) or shared (connected spot) across different samples. (**c**) Numbers of unique or shared CHON formulas. Only the 20 largest exclusive intersections are shown, ranked in descending order by the number of distinct CHON formulas. Each formula is assigned to one intersection according to its exact detection pattern across the nine samples. (**d**) The pie chart shows the distribution of the seven subgroups among the 407 CHON formulas shared by all samples. (**e**) Van Krevelen diagrams of CHON formulas common to all samples.

The overlap of molecular formulas in CHON among reservoir samples is presented in Figure 3. A total of 5081 distinct CHON molecular formulas were detected across the nine samples. Of these, 407 were detected in all nine samples, 2158 were detected in only one sample, and the remaining 2516 were detected in two to eight samples. The numbers of sample-specific formulas ranked in the order NAAN, PXZ, LGZ, SHTJ, SJZ, MJ, SHXS, DG, and ZLZ. Among the molecular formulas shared by all samples, region E compounds were the most abundant (77.8%), followed by region B compounds (12.0%).

**Figure 4:**
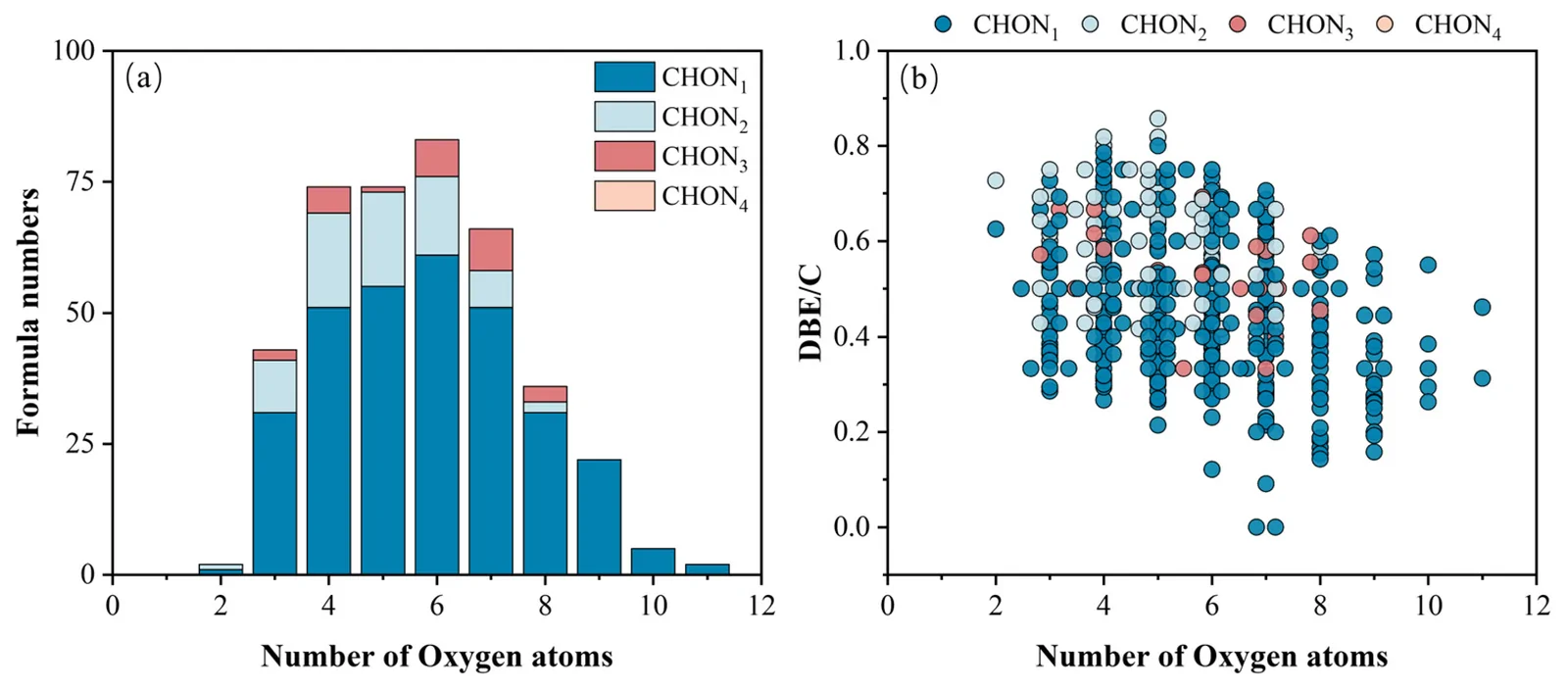
(**a**) Oxygen distribution for CHON formulas shared by all samples. (**b**) Double-bond equivalent (DBE) normalized by carbon number vs. number of oxygen atoms for shared CHON formulas. Overlapping points were slightly jittered to improve visibility.

The distributions of CHON formulas shared by all samples in terms of oxygen atom number and DBE/C are presented in Figure 4. Based on oxygen atom number, the shared CHON formulas were classified into ten subgroups, including O_x_N_1_ (O_2_N_1_-O_11_N_1_), O_x_N_2_ (O_2_N_2_-O_8_N_2_), and O_x_N_3_ (O_3_N_3_-O_8_N_3_). Among these subgroups, CHON_1_ was dominant throughout, whereas CHON_4_ was not detected among the shared molecular formulas. Most shared formulas were distributed in the O_4_N_y_ to O_6_N_y_ range and were mainly composed of molecules with low nitrogen content. To minimize the influence of carbon number on molecular formulas, DBE was normalized to carbon number as DBE/C. The results showed that the DBE/C values of the shared formulas were mainly concentrated between 0.3 and 0.8. CHON_1_ formulas showed a broad distribution of DBE/C, reaching the widest range when O = 7. The DBE/C of CHON_1_ generally decreased as the number of O atoms increased. In contrast, CHON_2_ and CHON_3_ were mainly concentrated in the low-oxygen-content and high-DBE/C regions, indicating a higher degree of unsaturation and a greater contribution of aromatic structures. Among the 407 shared CHON formulas, 49 (12.0%) fell within the peptide-like region defined in Section 2.3 and Table S2. Of these, 45 contained one nitrogen atom, with O_7_N_1_-O_9_N_1_ accounting for 31 formulas (63.3% of the peptide-like subset). Peptide-like formulas also occurred in O_5_N_2_, O_6_N_3_, and O_7_N_3_ (Table S10).

### 2.4. Characterization of Sample-Specific CHON Formulas

**Figure 5:**
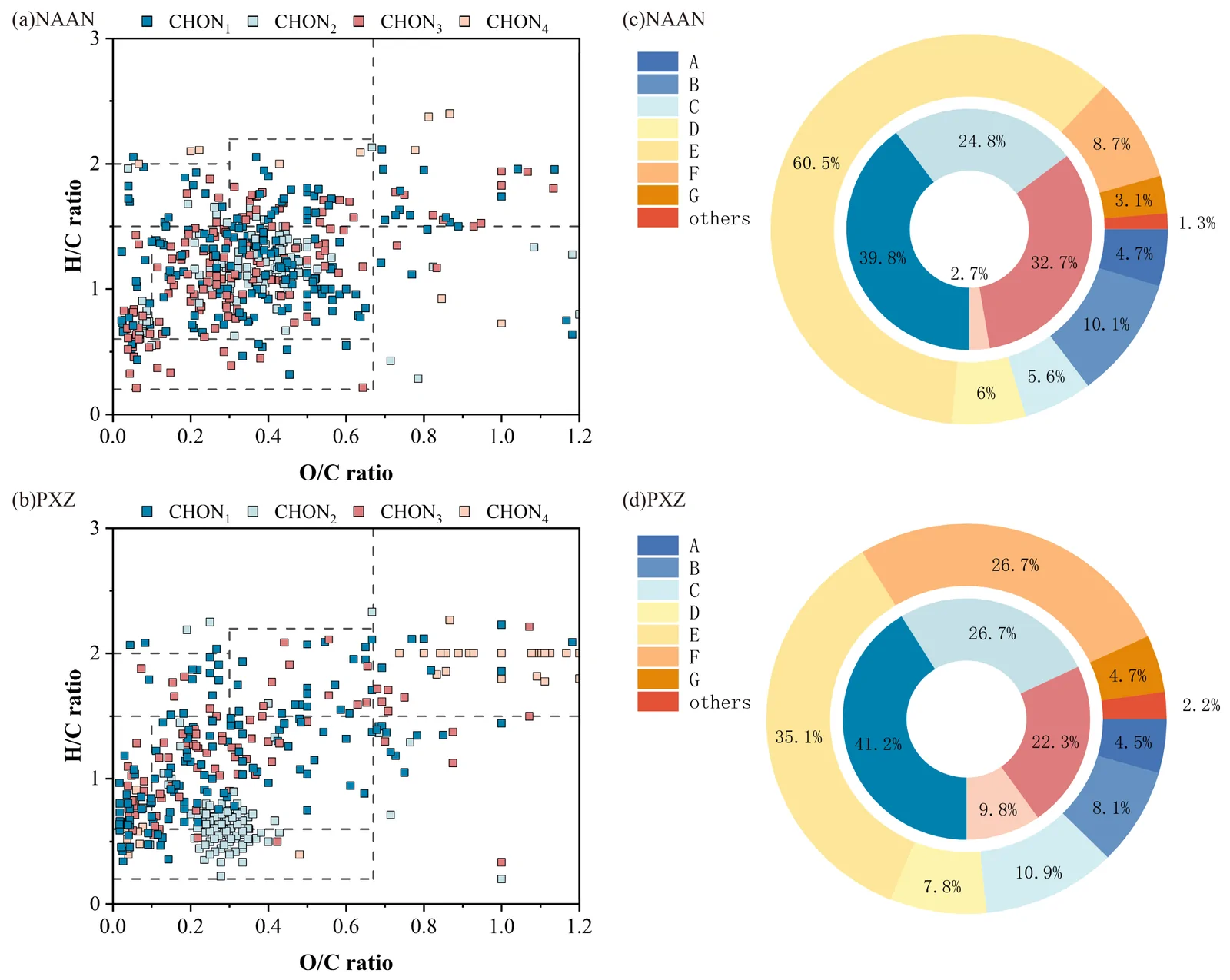
Van Krevelen diagram of sample-specific CHON formulas in the NAAN and PXZ (**a**,**b**). Relative abundances of CHON classes (inner ring) and Bianco subgroups (outer ring) for sample-specific CHON formulas from NAAN and PXZ (**c**,**d**).

The VK distributions and Bianco classifications of unique CHON formulas at each sample are presented in Figure 5 and Figures S5 and S6. Most formulas were widely distributed across the VK diagrams. Across all samples, unique CHON formulas were dominated by region E, although its proportion varied considerably. The highest fraction of region E was found at NAAN (60.40%) and the lowest at PXZ (35.10%). In addition to region E, region F showed marked variation among samples, reaching 26.74% at PXZ and 20.16% at DG. The nitrogen content of unique CHON formulas also differed among samples but was generally dominated by CHON_1_ and CHON_3_. These two groups contributed similarly in NAAN, SHXS, DG, and LGZ, whereas CHON_1_ dominated in SHTJ, PXZ, MJ, and SJZ, and CHON_3_ dominated in ZLZ. Moreover, the proportion of CHON_2_ increased from ~10% to ~20% at NAAN, SHTJ, SHXS, and PXZ, while CHON_4_ consistently accounted for only a minor fraction in all samples. CHON_2_ formulas in PXZ were mainly concentrated in the range of 0.2 < H/C < 1.0 and 0.2 < O/C < 0.4, whereas those in LGZ were mainly in 2.0 < H/C < 2.2 and 0.5 < O/C < 0.8; both differed from the other samples. These results indicate that there are clear spatial differences in nitrogen composition and molecular characteristics of DON between the northern and southern regions of the Beidagang Reservoir.

## 3. Discussion

### 3.1. Correlation Between Chemical Properties and Molecular Composition

The results in the previous section illustrate that DOC concentrations in the Beidagang Reservoir were relatively stable. In contrast, DON and TDN exhibited more pronounced spatial variability, suggesting distinct migration and transformation behaviors between organic carbon and organic nitrogen. CRAMs are recognized as important components of aquatic DOM, characterized by carboxyl-rich alicyclic structures and generally low biodegradability [19]. The relative stability of DOC may be attributed to the dominance of CRAM-like structures within DOM. However, CRAM-like compounds should not be simply regarded as refractory end products. CRAMs with lower O/C and higher H/C ratios are more susceptible to microbial processing and environmental transformation [20]. VK diagram analysis showed a higher proportion of CRAM-like formulas with lower O/C and higher H/C ratios at the northern and upstream sites. This pattern indicates spatial differences in the elemental composition of CRAM-like formulas. However, these ratios alone cannot establish relative biodegradability or distinguish isomeric structures. The role of microbial processing remains unresolved because no microbial degradation experiments were performed.

Compared with DOC, the spatial patterns of DON and other nitrogen indices more directly reflected nitrogen transformation processes within the reservoir. DON can serve as both a bioavailable substrate for microorganisms and a precursor of more recalcitrant nitrogen-containing compounds [2]. In this study, DON concentrations varied markedly among sampling sites. For example, upstream sites, NAAN and DG, showed high concentrations where external organic nitrogen inputs may have contributed to the observed enrichment, whereas NH_4_^+^-N showed no clear spatial trend, except for relatively elevated levels at PXZ. This pattern may be related to the relatively comparable proportions of labile compounds, such as lipid-like, aliphatic/peptide-like, and carbohydrate-like formulas. In general, DON concentrations tended to be higher in the upstream region and lower in the downstream region, suggesting that DON was progressively transformed into other DON fractions and inorganic nitrogen or assimilated by phytoplankton and bacteria during transport [3,21]. Signal intensities were not used to quantify molecular abundance differences among sites because they also depend on ionization response and sample matrix effects. Future work will incorporate normalized intensity comparisons and inter-sample ranking to further examine spatial differences in DON composition.

Water types were determined based on the equivalent percentage of major ions (Table S7). The results indicated that the Beidagang Reservoir was predominantly characterized by a Na-Cl water type, with additional influences from multiple geochemical processes. NAAN, ZLZ, MJ and DG showed higher proportions of Mg^2+^ and Ca^2+^, reflecting contributions from silicate weathering. Elevated SO_4_^2−^ proportions at NAAN, SHXS, LGZ, and SJZ suggest inputs from sulfur-related processes, while higher HCO_3_^−^ proportions at ZLZ, DG, and SJZ indicate the influence of carbonate weathering. At MJ, HCO_3_^−^ dominated the anion composition instead of Cl^−^, implying a weaker impact of salinization. Notably, SO_4_^2−^ concentrations did not exhibit a consistent relationship with the abundance of sulfur-containing molecular formulas, indicating that sulfur-containing DOM is influenced by multiple processes rather than solely controlled by sulfate availability.

### 3.2. Shared Molecular Features of Reservoir DON

Shared molecular formulas may reflect similar sources and common transformation processes of dissolved organic matter across samples [22]. Figure 3 indicates that peptide-like compounds were also identified as major components of DON in the Beidagang Reservoir besides CRAM-like structures. Here, “peptide-like” denotes a compositional category rather than confirmed peptide structures. Previous studies have examined potential peptide-related structures using peptide databases [23] and LC-FT-ICR MS with candidate-structure screening [24]. Although aliphatic/peptide-like compounds are generally considered relatively bioavailable and can be utilized by algae and microorganisms, they were widely detected across all samples in this study. This occurrence may reflect common inputs, internal production, or persistence within the reservoir. Oxidative dearomatization can promote the formation of oxygenated aliphatic compounds in aquatic environments [25]. This process may contribute to the occurrence of peptide-like formulas.

Molecular formulas with low nitrogen and moderate oxygen levels were shared among all samples of Beidagang Reservoir. However, molecular formulas with higher nitrogen levels (CHON_4_) did not constitute a common or stable fraction. This may be partly attributed to the low abundance of CHON_4_ (0.6–2.9%), which introduces greater uncertainty in their detection across all samples. Although DBE/C may not fully represent C=C unsaturation because of N-C bonding in CHON compounds, it still remains a useful indicator for assessing molecular saturation and aromaticity [26]. The shared DON molecular formulas in the Beidagang Reservoir were therefore dominated by moderately unsaturated structures. In addition, the low-nitrogen formulas (CHON_1_) likely encompassed different types of structural forms with varying degrees of unsaturation, whereas high-nitrogen formulas generally exhibited low-oxygen structures and a high degree of unsaturation. The O_x_N_y_ analysis further showed that shared peptide-like formulas were predominantly associated with N_1_ classes, particularly O_7_N_1_-O_9_N_1_. However, these classes also contained formulas outside the peptide-like region, indicating compositional heterogeneity within individual O_x_N_y_ classes.

### 3.3. Sample-Specific Molecular Features of Reservoir DON

Compared with the CHON formulas shared by all samples, the sample-specific CHON formulas displayed clear differences in the relative proportions of molecular classes and nitrogen atom numbers among sampling sites (Figure 5 and Figures S5 and S6). These differences suggested that DON in different regions of the Beidagang Reservoir may have distinct sources and formation pathways. The sample-specific molecular signatures may be associated with both local human activities and the geographic location of each sampling site.

Sample-specific formulas were generally dominated by CRAM-like structures in Beidagang Reservoir, but their distributions differed among sampling sites. For example, NAAN had a higher proportion of CRAM-like structures than the other samples. This pattern may be partly associated with its location in the northern upstream area of the reservoir, adjacent to the wetland nature reserve, where local human disturbance is relatively weak and external DON inputs may be limited. In addition, a fraction of microbially labile DOM may be decomposed and subsequently transformed into CRAM-like structures [27,28]. PXZ showed the lowest proportion of CRAM-like structures, accompanied by higher contributions of aromatic structures and highly oxygenated compounds than NAAN. This pattern may be related to the downstream location of PXZ in the northern part of the reservoir, where DON molecules may undergo molecular transformation during transport, leading to the formation or accumulation of more aromatic structures. In addition, PXZ is close to the Dagang Oilfield, so aromatic-structure compounds may be detected because of atmospheric deposition. DG also showed a relatively high proportion of aromatic structures, whereas the contribution of CRAM-like compounds did not decrease markedly. This may be because DG is located in the upstream part of the reservoir but is closer than NAAN to areas affected by human activities.

CHON_1_ and CHON_3_ made relatively high contributions to the sample-specific formulas in the Beidagang Reservoir, whereas CHON_2_ may represent an intermediate group involved in denitrogenation or nitrogen-addition reactions [3,7]. The H/C and O/C ratios of CHON_2_ formulas showed clear clustering patterns in some samples. For example, in PXZ, CHON_2_ formulas were mainly distributed within the CRAM-like and aromatic structure regions, whereas in LGZ, they were concentrated in the peptide-like and carbohydrate-like regions. These results suggest that CHON_2_ molecules at different sampling sites may reflect structural differences. However, their specific structures and formation pathways remain unresolved.

### 3.4. Possible Precursor–Product Pairs Along the Flow Direction

**Figure 6:**
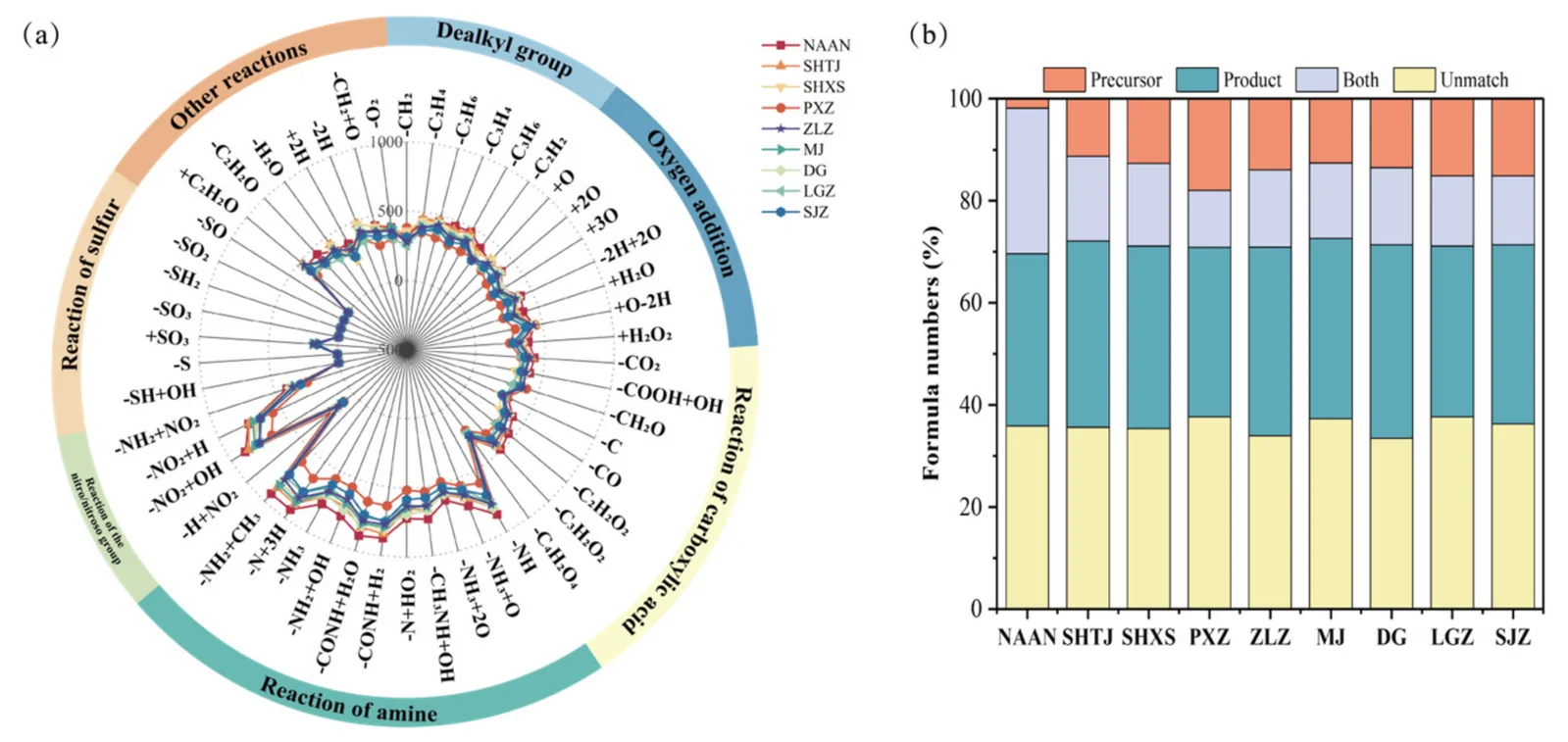
(**a**) Number of possible transformation reactions along the flow direction starting from NAAN. (**b**) Distribution of the formula number involved in the possible transformation reactions.

To further examine the potential transformation processes of DON in the Beidagang Reservoir from the perspective of molecular reaction characteristics, CHON formulas detected at the upstream site NAAN were used as putative precursors, whereas all assigned formulas in each reservoir sample were treated as possible products. Precursor–product relationships were identified based on accurate m/z differences, and the number of matched reactions was calculated for each sample (Figure 6a). According to the matching results, molecular formulas were further classified into four categories: precursor-only, product-only, both precursor and product, and unmatched formulas (Figure 6b). The matched reactions were grouped into demethylation, oxygenation, decarboxylation, amination, nitrosation, sulfur-related reactions, and other reactions, which represent common molecular transformation pathways during radical oxidation and photochemical processing [22,29,30]. Detailed information on possible reactions is provided in the Supporting Information (Table S8).

The DON transformation reactions showed similar trends among different regions of the Beidagang Reservoir. These results indicate that FT-ICR MS can identify potential precursor–product relationships at the molecular level and provide useful information on possible DON transformation pathways. Approximately 60% of the DON formulas were involved in at least one putative precursor–product pair under the matching rules used in this study, and product-only formulas dominated at all sampling sites. Under the matching rules used in this study, NAAN showed a markedly lower proportion of precursor-only formulas than the other sites, but a higher proportion of formulas that acted as both precursors and products. Among all putative precursor–product pairs, approximately 40% were assigned to the amination category, identifying nitrogen-related transformation hypotheses for further investigation. This pattern suggests that transformations of nitrogen functional groups may represent a major pathway for DON molecular conversion along the reservoir flow direction [31]. In contrast, sulfur compound reactions accounted for the smallest proportion, indicating that the incorporation of sulfur functional groups was not a dominant process during DON transformation. This may be partly explained by the relatively high chemical inertness of some organic sulfur compounds in aquatic environments [32]. In addition, the matching strategy used CHON formulas from NAAN as the initial precursor pool; reactions involving the removal of sulfur functional groups were not detected.

Mass-difference matching analysis was used to explore potential DON transformation relationships among reservoir samples. CHON formulas from NAAN were selected as putative precursors because NAAN is located upstream, providing a reference for comparisons along the flow direction. However, this selected precursor pool does not include all possible precursors from other inputs or internal reservoir processes. The matched pairs also depend on the reference transformation categories and matching tolerance. These results therefore indicate putative transformation relationships rather than confirmed reactions. Additional experimental evidence is needed to verify specific reactions and their directions.

### 3.5. Possible Sources of Reservoir DON

**Figure 7:**
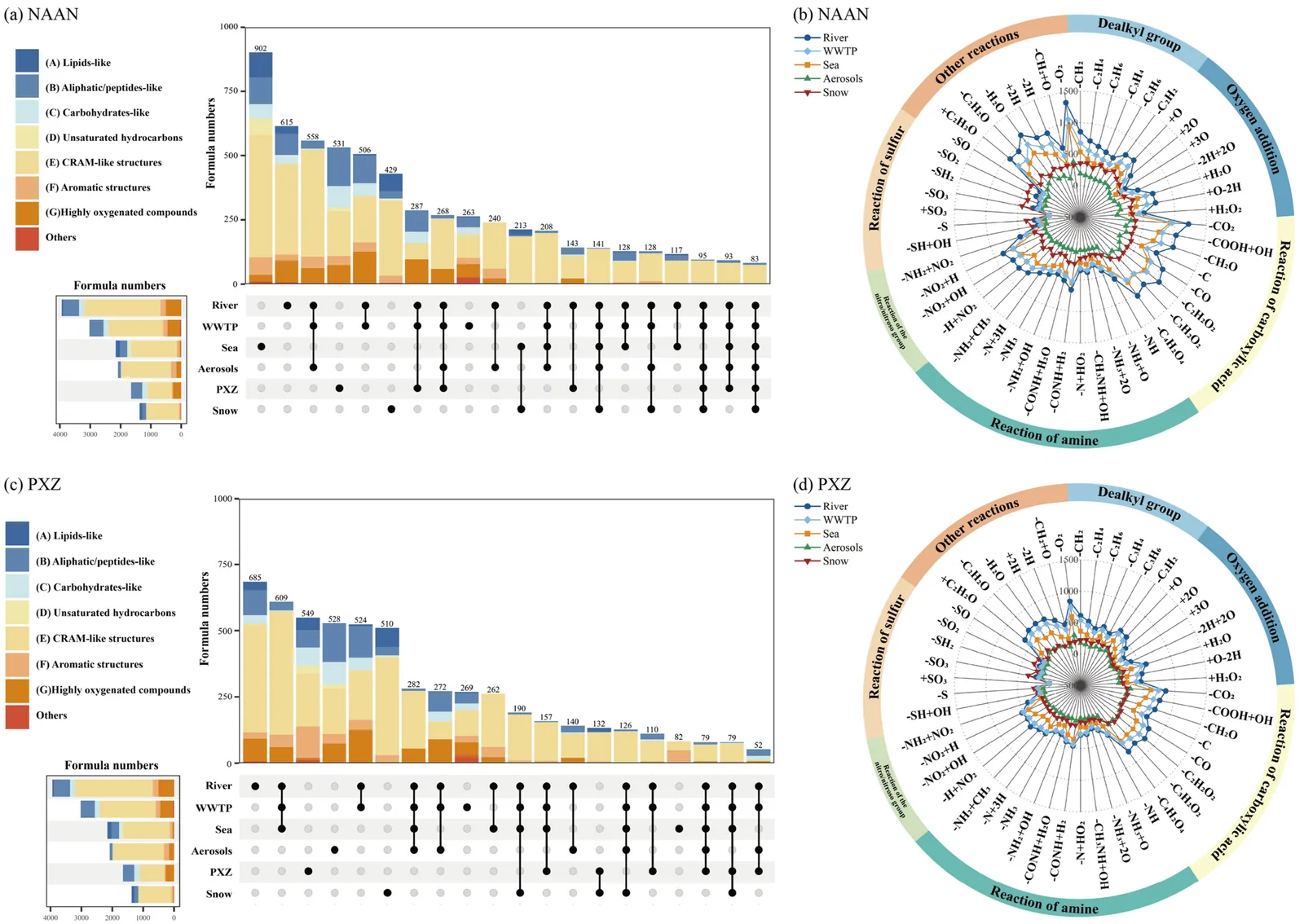
Shared and unique CHON formula numbers between NAAN (**a**) or PXZ (**c**) and the reference samples (river, WWTP, sea, aerosols, and snow). Only the top 20 intersections ranked by formula counts are shown. Numbers of possible transformation reactions between NAAN (**b**) or PXZ (**d**) and the reference samples.

To explore the potential sources of DON in the Beidagang Reservoir, reference samples from different aquatic and atmospheric environments (river water, wastewater, seawater, aerosols, and snow) were selected based on previous studies [14,16,33,34]. These samples included river water, municipal wastewater, seawater, aerosols, and snow. The shared molecular formulas and putative transformation relationships were then compared between the reference samples the upstream site (NAAN) or the downstream site (PXZ) (Figure 7). In the mass-difference matching analysis, molecular formulas from the reference samples were treated as putative precursors, whereas CHON formulas detected in the reservoir samples were regarded as possible products. Detailed information on the reference samples is provided in the Supporting Information (Table S9).

Among the 2155 DON formulas detected at NAAN, 902 formulas were unique to this sample, accounting for 41.86% of the total DON formulas (Figure 7a). This relatively high proportion of unique formulas suggests that upstream DON was influenced not only by external inputs, such as surface water inflow and atmospheric deposition, but also internal processes, including microbial activity, molecular transformation and local climate conditions. In addition to unique formulas, several high-frequency intersections were observed between NAAN and the reference samples. Specifically, 213 formulas were shared between NAAN and snow; 208 formulas were shared among NAAN, river water, wastewater, and seawater; and 141 formulas were common to NAAN, river water, wastewater, seawater, and snow. These intersections indicate that upstream DON in the Beidagang Reservoir may be influenced by multiple environmental media, with surface runoff, anthropogenic inputs, and atmospheric deposition. In addition, 83 formulas were detected in all samples, suggesting that these formulas may represent widely occurring molecular components across the compared environmental matrices. Other intersections were also present but contained fewer formulas and are therefore not shown in the figure.

The number of potential reaction pairs between all formulas at reference samples and CHON formulas at NAAN differed markedly among sources, following the order river water > wastewater > seawater > snow > aerosols (Figure 7b). This pattern suggests that DON derived from surface runoff and anthropogenic inputs may serve as important precursor pools for reservoir DON. Among different reaction types, reactions of carboxylic acids were the most abundant across all precursor–product pairs, accounting for approximately 19.86–30.45% of the total matched reactions. This result suggests that carboxyl-related transformations may be worth further investigation in the upstream region of the Beidagang Reservoir, consistent with previous findings [31]. Other reactions, including deoxygenation, dehydration, hydrogenation, small-molecule addition or loss, and dealkylation, showed comparable contributions, generally accounting for approximately 10–25% of the total reactions. Notably, organic matter from aerosols showed a much lower number of amine reactions than the other reference sources, whereas organic matter from snow showed a higher number of sulfur-related reactions.

Among the 1395 DON formulas detected at PXZ, 549 formulas were unique, accounting for 39.35% of the total DON formulas (Figure 7c). High-frequency intersections between PXZ and the reference samples included 157 formulas shared among PXZ, river water, wastewater, and seawater; 132 formulas shared between PXZ and snow; and 110 formulas shared among PXZ, river water, and wastewater. These intersections indicated that downstream DON in the Beidagang Reservoir may also originate from multiple environmental sources and be influenced by surface runoff, anthropogenic inputs, and seawater-related molecular signatures. The number of PXZ formulas shared with wastewater and with seawater was higher than that of NAAN formulas. This indicates greater molecular overlap with the wastewater and seawater reference samples, consistent with possible links to these sources.

The number of putative reaction pairs between all formulas at reference samples and CHON formulas at PXZ was lower than that at NAAN, although the overall trend was similar (Figure 7d). In the downstream sample, wastewater and aerosol reference samples showed higher proportions of matched pairs than in the upstream sample, whereas the proportion associated with snow was lower. This pattern suggests that downstream DON molecular composition may be more strongly influenced by anthropogenic inputs and atmospheric particulate matter, while the influence of wet deposition may be weakened. Among all potential reaction types, reactions of carboxylic acids still remained the dominant reaction type, accounting for approximately 21.58–29.89% of the total matched reactions. The proportions of other reaction types exhibited no clear difference from those observed in the upstream sample.

Overall, molecular comparisons suggest possible links between reservoir DON and atmospheric deposition, surface runoff, anthropogenic inputs, and seawater-related sources. These associations differed between upstream and downstream samples. After external DON enters the reservoir, its molecular composition may undergo substantial transformation, with reactions of carboxylic acids representing one important putative pathway. In particular, the strong molecular overlap and matches of precursor–product pairs between reservoir DON and reference samples of river water or wastewater reference samples suggested possible links with surface runoff and human activities.

## 4. Materials and Methods

### 4.1. Site Description and Sample Selection

Nine water samples (NAAN, SHTJ, SHXS, PXZ, ZLZ, MJ, DG, LGZ, SJZ) were collected in October 2024 from the Beidagang Reservoir (38°50′06″ N, 117°28′12″ E). Sampling sites were classified by geographic position and relative position along the schematic water flow path (Figure S1). The northern sites include NAAN, SHXS, SHTJ, and PXZ, whereas the southern sites include ZLZ, MJ, DG, LGZ, and SJZ. Although DG occupies an intermediate north–south position, it is not adjacent to the Beidagang Wetland Nature Reserve and was therefore included in the southern group. Along the indicated flow path, NAAN, ZLZ, MJ, DG, and LGZ were classified as upstream sites, whereas SJZ, SHTJ, SHXS, and PXZ were classified as downstream sites. The two classifications describe different spatial features and therefore overlap.

The Beidagang Reservoir is located in the warm-temperate monsoon region, and water generally flows from west to east. It should be noted that the northern part of the reservoir is likely less impacted by human activity, as it borders the Beidagang Wetland Nature Reserve. The samples were collected using pre-cleaned HDPE bottles rinsed with Milli-Q water. Then the samples were transported to the laboratory within 5 h. All samples and blank samples were filtered through 0.7 μm glass fiber filters (Whatman GFF, Whatman, Maidstone, U.K.; combusted for 4 h at 450 °C) within 24 h.

### 4.2. FT-ICR MS Analysis

Water samples were extracted using solid-phase extraction cartridges (200 mg, Oasis HLB, 6 cc, Waters, Milford, MA, USA) and analyzed by a 7.0 T superconducting magnet Bruker Solarix FT-ICR MS (SolariX 2xR, Bruker, Bremen, Germany), which was equipped with an electrospray ionization source (ESI) in the negative ion mode. Negative-mode ESI was used because it favors the detection of molecules bearing deprotonatable acidic functional groups and is widely applied in aquatic DOM characterization [35,36]. The molecular results presented here may have limited the detection of some nitrogen-containing compounds, particularly amines that are more efficiently ionized in positive mode. Water samples were introduced into the instrument by continuous infusion at 150 µL h^−1^, with a capillary voltage of 5.0 kV. Mass spectra were acquired over an m/z range of 150–1000 Da. Further details on the extraction procedure and instrument parameters are provided in previous studies [33] and the Supporting Information.

### 4.3. Molecular Formula Assignment

After FT-ICR MS acquisition, raw spectra were internally recalibrated in DataAnalysis (version 5.0, Bruker Daltonics, Bremen, Germany). Molecular formulas were assigned using Formularity (version 1.0), developed by Andrey Liyu and Nikola Tolic at Pacific Northwest National Laboratory (PNNL, Richland, WA, USA) [37]. Paired mass distance analysis was performed using a custom R script associated with the method described by Bingliang Zhang [11]. Mass-difference matching analysis was used to identify putative transformation relationships between assigned molecular formulas by matching their mass differences to theoretical mass differences associated with predefined net elemental changes [11,38]. The reference transformation categories are listed in Table S8. A matching formula pair was interpreted as a putative precursor–product pair, rather than evidence of an experimentally confirmed reaction.

Elemental compositions, among the m/z range of 150–800 Da, were assigned a signal-to-noise ratio of at least 4 and a mass tolerance of ±0.5 ppm. The elemental restrictions of the samples were set to atom numbers of ^12^C (1–60), ^1^H (1–100), ^16^O (1–30), ^14^N (0–4), and ^32^S (0–2). The isotopomers were not considered in the present study. Peaks observed in sample blanks and reagent blanks with an average S/N greater than 10 were removed from the final peak list for all samples [39].

Definitions and calculation procedures of the commonly used indices for each molecular formula (C_c_H_h_O_o_N_n_S_s_) are provided in the Supporting Information. Reported mean molecular descriptors were calculated as arithmetic means across the assigned molecular formulas in each sample or elemental group. Van Krevelen (VK) diagrams were used to visualize molecular composition based on H/C and O/C ratios [40]. Molecular formulas were grouped into seven classes depending on their H/C and O/C ratios: (A) lipid-like, (B) aliphatic/peptide-like, (C) carbohydrate-like, (D) unsaturated hydrocarbons, (E) carboxylic-rich alicyclic molecule (CRAM)-like, (F) aromatic structures, and (G) highly oxygenated compounds (HOCs) [18]. These categories describe H/C- and O/C-based compositional regions rather than confirmed molecular structures. Within region B, nitrogen-free formulas (CHO and CHOS) were classified as aliphatic-like, whereas nitrogen-containing formulas (CHON and CHONS) were classified as peptide-like [41,42]. The H/C and O/C boundaries are provided in Table S2. Nitrogen-containing formulas in this region may also represent non-peptide isomers. The nominal oxidation state of carbon (NOSC) was calculated following LaRowe and Van Cappellen [43]. Double bond equivalents (DBE) provide a quantitative measure of the degree of unsaturation of individual molecules and can be used to distinguish structurally similar isomers [44].

For the upset plot analysis, duplicate records of the same molecular formula within each sample were removed, and a presence–absence matrix was constructed to identify exclusive intersections across the nine samples.

### 4.4. Analyses of DOC, DON and Inorganic Ions

DOC was measured by a TOC analyzer (TOC-5000; Shanghai Metash Instrument Co., Ltd., Shanghai, China). TDN, NH_4_^+^, and NO_3_^−^ were acquired by a UV-vis spectrophotometer (TU-1810; Beijing Puxi General Instrument Co., Ltd., Beijing, China) without continuous flow analysis. TDN was determined after alkaline potassium persulfate digestion, followed by UV measurement of the resulting nitrate. Ammonium was determined using Nessler’s reagent, and nitrate was determined by UV spectrophotometry. Detailed procedures are provided in the Supporting Information. DON was calculated as the difference between TDN and the sum of inorganic N (short for DIN, including NO_3_^−^ and NH_4_^+^). NO_2_^−^ was below the UV-vis detection limit and was therefore ignored. All nitrogen concentrations were expressed as mg N L^−1^ before calculating DON. Cations (Na^+^, Ca^2+^, and Mg^2+^) and anions (Cl^−^ and SO_4_^2−^) in filtered samples (0.22 μm MCE filters) were measured by ion chromatography (PIC-10; Qingdao Puren Instrument Co., Ltd., Qingdao, China). Details about the analytical procedures are described in the Supporting Information.

## 5. Conclusions

The Beidagang Reservoir is an important water conservancy facility in Tianjin and plays an important role in urban water supply. Based on field monitoring data and FT-ICR MS, this study revealed the molecular composition and spatial distribution of DON in the Beidagang Reservoir. The molecular findings apply to the solid-phase-extractable fraction detectable by negative-mode ESI. The reservoir DON exhibited pronounced spatial variability and chemical diversity, and its molecular composition was likely influenced by both external inputs and internal transformation processes. Our results suggest that DON molecules may undergo continuous transformation during transport within the reservoir, with nitrogen functional group transformations playing an important role in this process. In addition, source-related analysis based on accurate mass-difference matching demonstrated that there are possible links between upstream DON and the influences of surface runoff, atmospheric deposition, and internal transformation processes, whereas there are stronger possible links between downstream DON and anthropogenic activities and seawater-related sources. Overall, the molecular composition and transformation of DON in the Beidagang Reservoir are controlled by the combined effects of external inputs and internal biogeochemical processes.

## Data Availability

All data that support the findings of this study are available within the article and its Supplementary Materials.

## Supplementary Materials

The following supporting information can be downloaded at https://www.mdpi.com/article/10.3390/molecules31203774/s1, S1 includes Supplementary Materials and methods, Supplementary results, Tables S1–S10, and Figures S1–S15. S2 is an Excel workbook containing the complete assigned molecular formula datasets for all nine water samples, including elemental compositions, elemental group classifications, measured m/z values, peak heights, signal-to-noise ratios, resolving powers, and reported intensity percentages. A separate worksheet contains the 407 CHON molecular formulas shared among all nine samples, together with their elemental compositions, O/C and H/C ratios. Each sample-specific worksheet is named according to the corresponding sample ID, and the worksheet about shared CHON formulas is named “shared CHON formulas of 9 water samples”. Table S1: The concentrations of DOC and nitrogen indices (mg L^−1^) of the water samples. Table S2: Stoichiometric boundaries of Van Krevelen classes. Table S3: The concentrations of inorganic ions (meq L^−1^) of the water samples. Table S4: The average composition properties of water samples. Table S5: Comparison of chemical characteristics of organic compounds in water samples by FT-ICR MS in the negative ESI mode. Table S6: Formula number percentage (%) of nine water samples in the BDG reservoir. Table S7: The hydrochemical classification of samples in the Beidagang Reservoir. Table S8: Summary of the possible transformation reactions in aquatic ecosystems. Table S9: Information about reference samples used to trace possible source. Table S10: Peptide-like formulas within CHON O_x_N_y_ classes shared by all nine samples. Figure S1: Water-sampling sites for this work in the Beidagang Reservoir (Tianjin, China). Figure S2: Concentrations of organic matter and inorganic ions. Figure S3: Reconstructed Fourier-transform ion cyclotron resonance mass spectra for the nine water samples. Figure S4: Statistical distribution of CHO, CHON, CHOS, and CHONS formulas across water samples. Figure S5: Van Krevelen diagrams (a–d), relative abundances and Bianco subgroups (e–h) of sample-specific CHON formulas in SHTJ, SHXS, ZLZ, MJ. Figure S6: Van Krevelen diagrams (a–c), relative abundances and Bianco subgroups (d–f) of sample-specific CHON formulas in LGZ, DG, SJZ. Figure S7: Heatmap of molecular subgroup percentages across sampling sites in the Beidagang Reservoir. Figure S8: Van Krevelen diagrams (a) and NOSC via carbon number (b) of assigned formulas in SHTJ. Figure S9: Van Krevelen diagrams (a) and NOSC via carbon number (b) of assigned formulas in SHXS. Figure S10: Van Krevelen diagrams (a) and NOSC via carbon number (b) of assigned formulas in ZLZ. Figure S11: Van Krevelen diagrams (a) and NOSC via carbon number (b) of assigned formulas in MJ. Figure S12: Van Krevelen diagrams (a) and NOSC via carbon number (b) of assigned formulas in LGZ. Figure S13: Van Krevelen diagrams (a) and NOSC via carbon number (b) of assigned formulas in DG. Figure S14: Van Krevelen diagrams (a) and NOSC via carbon number (b) of assigned formulas in SJZ. Figure S15: Van Krevelen diagrams (a) and NOSC via carbon number (b) of assigned formulas in PXZ.
