# Supplementary material for: Molecular-Level Insights into the Spatial Distribution, Transformation, and Potential Sources of Dissolved Organic Nitrogen in the Beidagang Reservoir
Source: Molecules. 2026 Oct 21;31(20):3774. doi: 10.3390/molecules31203774 (PMC13707603; doi:10.3390/molecules31203774)
Supplement: Supplementary file 1 [file molecules-31-03774-s001.zip › molecules-4574349-S1.pdf]

Supporting Information for

Molecular-Level Insights into the Spatial Distribution, Transformation, and Potential Sources of Dissolved Organic Nitrogen in the Beidagang Reservoir

1. Supplementary materials and methods

1.1 FT-ICR MS analysis

Filtered samples (250 mL) were extracted using solid-phase extraction (SPE) cartridges (Oasis HLB, 200 mg, 6 cc; Waters, USA). The cartridges were preconditioned with 5 mL methanol three times and 5 mL Milli-Q water twice. After sample loading, the cartridges were rinsed with 5 mL Milli-Q water to remove salts and then dried under a gentle stream for 1 h. Retained organic matter was eluted with 12 mL methanol. The eluate was immediately concentrated to 1 mL using a rotary evaporator and re-filtered through a 0.20 μm PTFE membrane. All extracts were stored at −20 °C until FT-ICR MS analysis.

For molecular formulas $C_cH_hO_oN_nS_s$, the nominal oxidation state of carbon (NOSC) was calculated following LaRowe and Van Cappellen [1]. For neutral formulas containing only C, H, O, N, and S, the equation is: NOSC = (2O-H+3N+2S)/C. Here, C, H, O, N, and S denote the atom numbers in the neutral molecular formula. Mean O/C, H/C, and NOSC values were calculated as arithmetic means across the assigned molecular formulas in each sample or elemental group, without intensity weighting. Double bond equivalents (DBE) provide a quantitative measure of the degree of unsaturation of individual molecules and can be used to distinguish structurally similar isomers[2]. The simplified equation is: DBE = 1+(2C-H+N)/2.

1.2 Analysis for DOC, DON and Inorganic Ions

DOC was measured by a TOC analyzer (TOC-5000; Shanghai Metash Instrument Co., Ltd., China). TDN, NH_4^+, and NO_3^- were acquired by a UV-vis spectrophotometer (TU-1810; Beijing Puxi General Instrument Co., Ltd., China). TDN was measured in filtered water samples using alkaline potassium persulfate digestion followed by UV spectrophotometry. Samples were digested at 120-124 °C for 30 min. After cooling, hydrochloric acid was added and the solutions were diluted to the specified volume. Absorbance was measured at 220 and 275 nm using 10 mm quartz cuvettes. The corrected absorbance was calculated as A220-2A275. Ammonium was determined using Nessler's reagent spectrophotometry. Potassium sodium tartrate and Nessler's reagent were added to the samples. After 10 min of color development, absorbance was measured at 420 nm. Nitrate was determined in undigested samples by UV spectrophotometry. Absorbance was measured at 220 and 275 nm using 10 mm quartz cuvettes, with correction calculated as A220-2A275. All measurements were performed using a TU-1810 UV-vis spectrophotometer without continuous flow analysis. Dissolved organic nitrogen (DON) was calculated as the difference between TDN and the sum of inorganic N (short for DIN, including NO_3^- and NH_4^+). NO_2^- was below the UV-vis detection limit and was therefore ignored. Cations (Na^+, Ca^{2+}, and Mg^{2+}) and anions (Cl^- and SO_4^{2-}) in filtered samples (0.22 μm MCE fliters) were measured by ion chromatography (PIC-10; Qingdao Puren Instrument Co., Ltd., China). HCO_3^- and CO_3^{2-} were acquired by titration, and concentration below the detection limit were ignored. Samples were diluted as needed to fall within the analytical range of each method. Reported concentrations were corrected using the corresponding dilution factors. Nitrate concentrations were converted from NO_3^- to NO_3^--N before calculating DON. All nitrogen concentrations are reported as mg N L^{-1}.

2. Supplementary results

The average O/C ratios of CHO, CHON, CHOS, and CHONS formulas were 0.31-0.33, 0.36-0.39, 0.36-0.41, and 0.36-0.44, respectively, while their average H/C ratios were 1.30-1.39, 1.25-1.29, 1.35-1.40, and 1.31-1.37, respectively (Table S5). CHON formulas generally exhibited lower H/C ratios than the other elemental groups, whereas sulfur-containing formulas showed relatively high O/C ratios. The mean nominal oxidation state of carbon (NOSC) ranged from -0.75 to -0.65 for CHO, -0.33 to -0.19 for CHON, -0.53 to -0.41 for CHOS, and -0.20 to 0.06 for CHONS formulas (Table S4). Mean NOSC followed the order CHONS > CHON > CHOS > CHO in all samples. Thus, sulfur-containing formulas showed both higher O/C ratios and higher mean NOSC than CHO formulas.

Hydrochemical classification showed that the northern reservoir samples were mainly characterized by Cl-Na or mixed Cl-SO_4 water types (Table S7). In contrast, several southern samples exhibited mixed HCO_3-Cl or HCO_3-dominated water types, particularly ZLZ, MJ, and DG. These classifications further demonstrate the spatial variability in the ionic composition of the Beidagang Reservoir.

3. Supplementary tables

Table S1. The concentration of DOC and nitrogen indices (mg L^{-1}) of the water samples.

Sample ID	DOC	TDN	DON	DIN	Ammonium	Nitrate
NAAN	9.32	21.75	18.67	3.08	1.98	1.10
SHTJ	15.63	21.90	20.24	1.66	1.21	0.45
SHXS	10.85	5.62	3.85	1.76	1.15	0.61
PXZ	10.30	7.72	4.18	3.54	2.28	1.26
ZLZ	10.12	5.41	2.24	3.17	1.47	1.71
MJ	10.59	8.03	4.06	2.55	1.64	0.92
DG	10.95	23.80	21.84	1.97	1.39	0.58
LGZ	9.60	4.83	3.13	1.70	1.11	0.59
SJZ	11.81	5.31	3.32	1.99	1.15	0.85
Mean	11.02	11.60	9.06	2.38	1.49	0.90

Table S2. Stoichiometric boundaries of Van Krevelen classes[3]. Within region B, formulas are further distinguished by nitrogen content in this study. Nitrogen-free formulas are termed aliphatic-like, whereas nitrogen-containing formulas are termed peptide-like [4,5].

	Class	O/C ratio	H/C ratio	Nitrogen criterion
A	Lipids-like	0≤ O/C≤0.3	1.5<H/C≤2.0	Not specified
B	Aliphatic-like	0.3<O/C≤0.67	1.5<H/C≤2.2	N = 0
	Peptide-like	0.3<O/C≤0.67	1.5<H/C≤2.2	N ≥ 1
C	Carbohydrates-like	0.67<O/C<1.0	1.5<H/C≤2.5	Not specified
D	Unsaturated hydrocarbons	O/C<0.1	0.67<H/C≤1.5	Not specified
E	CRAMs-like structures	0.1≤ O/C<0.67	0.67<H/C≤1.5	Not specified
F	Aromatic structures	O/C<0.67	0.2≤H/C≤0.67	Not specified
G	Highly oxygenated compounds	0.67≤O/C≤1.0	0.6<H/C≤1.5	Not specified
Others	Others	1.0<O/C	H/C<0.2, 2.5<H/C	Not specified

Table S3. The concentration of inorganic ions (meq L^{-1}) of the water samples.

Sample ID	Sodium	Magnesium	Calcium	Chloride	Sulfate	Bicarbonate	Summation
NAAN	4.35	3.87	2.53	10.34	10.92	4.68	36.70
SHTJ	22.56	6.85	3.20	31.17	5.98	1.04	72.19
SHXS	8.83	2.88	2.53	16.28	10.06	3.29	46.65
PXZ	12.68	5.53	5.47	24.92	9.66	4.16	65.18
ZLZ	4.85	2.35	3.87	4.36	2.62	3.99	24.11
MJ	6.86	1.95	4.13	1.49	0.70	4.51	21.03
DG	6.88	1.42	5.47	8.11	3.35	5.20	30.44
LGZ	9.66	3.67	3.87	8.91	7.05	3.12	38.36
SJZ	10.54	4.53	3.20	9.96	6.50	0.00	42.37
Mean	9.69	3.67	3.81	12.84	6.32	3.33	41.89

Table S4. The average composition properties in water samples. Mean NOS_C values were calculated as arithmetic means across the assigned molecular formulas in each elemental group.

Sample ID	Compounds	Formula numbers	Formula numbers (%)	NOS_C
NAAN	CHO	2342	37.05	-0.74
	CHON	2155	34.09	-0.27
	CHOS	1032	16.32	-0.52
	CHONS	793	12.54	-0.19
SHTJ	CHO	2018	35.00	-0.48
	CHON	1963	34.04	-0.74
	CHOS	1001	17.36	-0.33
	CHONS	784	13.60	-0.52
SHXS	CHO	1939	35.83	-0.17
	CHON	1818	33.59	-0.48
	CHOS	965	17.83	-0.75
	CHONS	690	12.75	-0.31
PXZ	CHO	1504	32.57	-0.52
	CHON	1395	30.21	-0.20
	CHOS	930	20.14	-0.49
	CHONS	789	17.09	-0.65
ZLZ	CHO	1802	34.62	-0.19
	CHON	1743	33.49	-0.41
	CHOS	876	16.83	0.06
	CHONS	784	15.06	-0.34
MJ	CHO	1897	33.22	-0.69
	CHON	1919	33.62	-0.24
	CHOS	1052	18.42	-0.42
	CHONS	842	14.74	-0.04
DG	CHO	1903	35.62	-0.40
	CHON	1791	33.53	-0.71
	CHOS	935	17.50	-0.26
	CHONS	713	13.35	-0.48
LGZ	CHO	1629	30.70	-0.10
	CHON	1812	34.14	-0.43
	CHOS	1050	19.79	-0.67
	CHONS	816	15.38	-0.25
SJZ	CHO	1797	34.54	-0.44
	CHON	1741	33.46	-0.10
	CHOS	952	18.30	-0.41
	CHONS	713	13.70	-0.68

Table S5. Comparison of chemical characteristics of organic compounds in water samples by FT-ICR MS in the negative ESI mode. Mean O/C and H/C ratios were calculated as arithmetic means across the assigned molecular formulas in each elemental group.

Sample ID	Compounds	O/C	H/C	Reference
NAAN	all	0.35	1.33	This work
SHTJ	all	0.35	1.34	This work
SHXS	all	0.35	1.35	This work
PXZ	all	0.38	1.31	This work
ZLZ	all	0.37	1.32	This work
MJ	all	0.36	1.32	This work
DG	all	0.37	1.31	This work
LGZ	all	0.36	1.32	This work
SJZ	all	0.36	1.34	This work
Aerosol	all	0.51	1.32	[6]
Snow	all	0.44	1.36	[7]
Marine water	all	0.52	1.18	[8]
River water	all	0.52	1.30	[9]
Wastewater	all	0.52	1.28	[9]
Rappbode Reservoir	all	0.51	1.18	
NAAN	CHO	0.32	1.39	This work
SHTJ	CHO	0.32	1.38	This work
SHXS	CHO	0.32	1.38	This work
PXZ	CHO	0.33	1.30	This work
ZLZ	CHO	0.32	1.33	This work
MJ	CHO	0.31	1.34	This work
DG	CHO	0.33	1.32	This work
LGZ	CHO	0.32	1.32	This work
SJZ	CHO	0.31	1.36	This work
Aerosol	CHO	0.51	1.26	[6]
Snow	CHO	0.43	1.28	[7]
Marine water	CHO	0.51	1.15	[8]
River water	CHO	0.50	1.30	[9]
Wastewater	CHO	0.50	1.22	[9]
Rappbode Reservoir	CHO	0.49	1.11	
NAAN	CHON	0.36	1.25	This work
SHTJ	CHON	0.36	1.29	This work
SHXS	CHON	0.36	1.29	This work
PXZ	CHON	0.39	1.27	This work
ZLZ	CHON	0.38	1.28	This work
MJ	CHON	0.37	1.27	This work
DG	CHON	0.38	1.27	This work
LGZ	CHON	0.37	1.27	This work

SJZ	CHON	0.36	1.29	This work
Aerosol	CHON	0.59	1.37	[6]
Snow	CHON	0.37	1.08	[7]
Marine water	CHON	0.50	1.10	[8]
River water	CHON	0.52	1.29	[9]
Wastewater	CHON	0.54	1.29	[9]
Rappbode Reservoir	CHON	0.51	1.16	
NAAN	CHOS	0.36	1.37	This work
SHTJ	CHOS	0.37	1.38	This work
SHXS	CHOS	0.38	1.40	This work
PXZ	CHOS	0.41	1.37	This work
ZLZ	CHOS	0.40	1.35	This work
MJ	CHOS	0.38	1.37	This work
DG	CHOS	0.40	1.36	This work
LGZ	CHOS	0.37	1.40	This work
SJZ	CHOS	0.38	1.39	This work
Aerosol	CHOS	0.49	1.47	[6]
Snow	CHOS	0.49	1.68	[7]
Marine water	CHOS	0.53	1.36	[8]
River water	CHOS	0.54	1.38	[9]
Wastewater	CHOS	0.49	1.36	[9]
Rappbode Reservoir	CHOS	0.53	1.34	
NAAN	CHONS	0.36	1.31	This work
SHTJ	CHONS	0.36	1.32	This work
SHXS	CHONS	0.36	1.33	This work
PXZ	CHONS	0.44	1.34	This work
ZLZ	CHONS	0.41	1.32	This work
MJ	CHONS	0.39	1.31	This work
DG	CHONS	0.39	1.31	This work
LGZ	CHONS	0.37	1.31	This work
SJZ	CHONS	0.42	1.37	This work
Aerosol	CHONS	0.38	1.19	[6]
Marine water	CHONS	0.57	1.26	[8]
River water	CHONS	0.53	1.19	[9]
Wastewater	CHONS	0.54	1.31	[9]
Rappbode Reservoir	CHONS	0.64	1.48	

Table S6. Formula number percentage (%) of nine water samples in BDG reservoir.

Sub-groups	Compou-nds	NAAN	SHTJ	SHXS	PXZ	ZLZ	MJ	DG	LGZ	SJZ
Lipids-like	CHO	20.71	20.56	21.20	14.16	16.98	18.29	17.60	20.01	21.26
	CHON	6.22	7.03	6.00	5.02	5.85	6.04	5.97	5.96	7.87
	CHOS	14.24	13.59	11.50	6.88	8.79	11.31	10.59	16.86	12.29
	CHONS	7.82	7.53	6.23	3.42	4.08	5.23	6.45	7.72	6.31
	All	13.10	12.97	12.45	8.10	9.93	10.96	10.99	12.70	13.09
Aliphatic/peptides-like*	CHO	15.20	15.01	15.16	11.57	12.71	12.76	12.03	11.54	12.35
	CHON	11.18	13.35	12.43	13.05	12.22	11.98	13.12	13.47	12.81
	CHOS	15.70	16.78	20.41	21.83	15.64	16.16	16.90	15.33	17.96
	CHONS	14.38	10.08	11.45	12.42	10.71	11.40	11.22	12.62	14.73
	All	13.81	14.08	14.71	14.23	12.74	12.92	13.14	13.11	13.86
Carbohydrates-like	CHO	0.43	0.10	0.00	0.93	0.17	0.05	0.11	0.06	0.11
	CHON	3.48	4.89	5.34	7.53	6.54	6.72	5.53	6.95	6.26
	CHOS	2.03	2.70	3.42	4.19	4.00	4.56	4.28	4.29	4.62
	CHONS	3.91	4.72	4.78	12.55	8.80	6.77	6.59	6.37	9.54
	All	2.17	2.81	3.01	5.57	4.25	4.11	3.52	4.22	4.29
Unsaturated hydrocarbons-like	CHO	1.67	2.28	2.53	2.33	2.94	3.27	2.47	2.58	3.45
	CHON	2.97	3.31	3.85	2.37	2.70	3.85	2.18	4.36	3.62
	CHOS	1.94	1.90	3.01	2.15	1.83	2.09	1.60	2.57	2.10
	CHONS	6.68	5.87	6.96	4.82	4.85	5.11	4.77	6.62	4.49
	All	2.78	3.05	3.62	2.73	2.96	3.52	2.53	3.81	3.40

CRAMs-like structures	CHO	59.95	60.01	59.05	67.95	64.93	63.36	65.42	62.86	60.77
	CHON	70.07	66.79	67.82	59.93	66.38	64.53	66.78	62.03	62.26
	CHOS	63.08	62.14	58.76	61.40	64.50	61.88	61.71	57.24	58.40
	CHONS	58.51	63.14	64.78	57.92	60.84	62.00	61.85	56.99	54.00
	All	63.78	63.11	62.68	62.49	64.73	63.28	64.75	60.56	59.91
Aromatic structures	CHO	1.62	1.73	1.86	2.86	1.83	2.00	2.05	2.52	1.78
	CHON	3.90	2.95	2.97	7.81	3.84	4.32	4.02	4.75	3.96
	CHOS	0.97	1.10	1.24	1.29	1.71	1.43	1.60	1.33	1.37
	CHONS	4.29	3.06	2.32	4.18	4.72	4.28	3.93	5.39	4.91
	All	2.63	2.22	2.18	4.27	2.92	3.01	2.88	3.49	2.86
Highly oxygenated compounds and others	CHO	0.43	0.30	0.21	0.20	0.44	0.26	0.32	0.43	0.28
	CHON	2.18	1.68	1.60	4.30	2.47	2.55	2.40	2.48	3.22
	CHOS	2.03	1.80	1.66	2.26	3.54	2.57	3.32	2.38	3.26
	CHONS	4.41	5.61	3.48	4.69	5.99	5.23	5.19	4.29	6.03
	All	1.74	1.75	1.35	2.62	2.48	2.19	2.19	2.11	2.59

*: Nitrogen-free formulas (CHO and CHOS) were classified as aliphatic-like, whereas nitrogen-containing formulas (CHON and CHONS) were classified as peptide-like.

Table S7. The hydrochemical classification of samples in Beidagang Reservoir

Samples	Hydrochemical classification
NAAN	$Cl{\cdot}SO_4$-Na·Mg
SHTJ	Cl-Na
SHXS	$Cl{\cdot}SO_4$-Na
PXZ	Cl-Na
ZLZ	$HCO_3{\cdot}Cl$-Na·Ca
MJ	HCO_3-Na·Ca
DG	$Cl{\cdot}HCO_3$-Na·Ca
LGZ	$Cl{\cdot}SO_4$-Na
SJZ	$Cl{\cdot}HCO_3{\cdot}SO_4$-Na

Table S8. Summary of the possible tranformation reactions in aquatic ecosystems[10-12].

NO.	Reaction	Formula difference	Reference
1	Dealkyl group	1-CH_2	[10]
2		1-C_2H_4	
3		1-C_2H_6	
4		1-C_3H_4	
5		1-C_3H_6	
6		1-C_2H_2	
7	Oxygen addition	1+O	
8		1+2O	
9		1+3O	
10		1-2H+2O	
11		1+H_2O	
12		1+O-2H	[12]
13		1+H_2O_2	
14	Reaction of carboxylic acid	1-CO_2	[10]
15		1-COOH+OH	
16		1-CH_2O	
17		1-C	
18		1-CO	[11]
19		1-$C_2H_2O_2$	[12]
20		1-$C_3H_2O_2$	
21		1-$C_4H_2O_4$	
22	Reaction of amine	1-NH	[10]
23		1-NH_3+O	
24		1-NH_3+2O	
25		1-CH_3NH+OH	
26		1-N+HO_2	
27		1-CONH+H_2	
28		1-CONH+H_2O	
29		1-NH_2+OH	[11]
30		1-NH_3	
31		1-N+3H	[12]
32		1-NH_2+CH_3	
33	Reaction of the nitro/nitroso group	1-H+NO_2	[10]
34		1-NO_2+OH	
35		1-NO_2+H	
36		1-NH_2+NO_2	[12]
37	Reaction of sulfur	1-SH+OH	[10]
38		1-S	
39		1+SO_3	
40		1-SO_3	
41		1-SH_2	

42		1-SO_2	
43		1-SO	[12]
44	Other reaction	1+C_2H_2O	[10]
45		1-C_2H_2O	
46		1-H_2O	
47		1+2H	
48		1-2H	
49		1-CH_2+O	[11]
50		1-O_2	[12]

Table S9. Information about reference samples used to trace possible source[6,9,13,14].

Sample	Location	Reference
River	Haihe River, China	[9]
WWTP	Tianjin, China	
Sea	Bohai Sea, China	[14]
Aerosol	Beijing, China	[6]
Snow	Tianjin, China	[13]

Table S10. Peptide-like formulas within CHON O_xN_y classes shared by all nine samples.

O_xN_y class	Total formula	Peptide-like formula	Peptide-like formula (%)
O_2N_1	1	0	0.00
O_3N_1	31	0	0.00
O_4N_1	51	2	3.90
O_5N_1	55	3	5.50
O_6N_1	61	6	9.80
O_7N_1	51	11	21.60
O_8N_1	31	10	32.30
O_9N_1	22	10	45.50
$O_{10}N_1$	5	3	60.00
$O_{11}N_1$	2	0	0.00
O_2N_2	1	0	0.00
O_3N_2	10	0	0.00
O_4N_2	18	0	0.00
O_5N_2	18	2	11.10
O_6N_2	15	0	0.00
O_7N_2	7	0	0.00
O_8N_2	2	0	0.00
O_3N_3	2	0	0.00
O_4N_3	5	0	0.00
O_5N_3	1	0	0.00
O_6N_3	7	1	14.30
O_7N_3	8	1	12.50
O_8N_3	3	0	0.00
Total	407	49	12.00

4. Supplementary figures

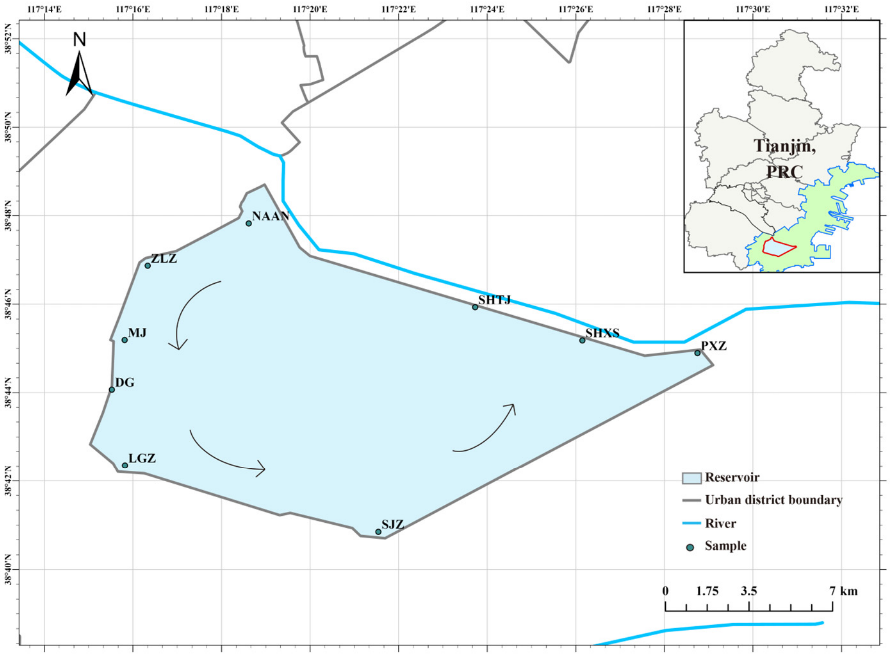

Figure S1. Water-sampling sites of this work in the Beidagang Reservoir (Tianjin, China). Black arrows indicate the schematic water flow direction used to explain the upstream/downstream classification.

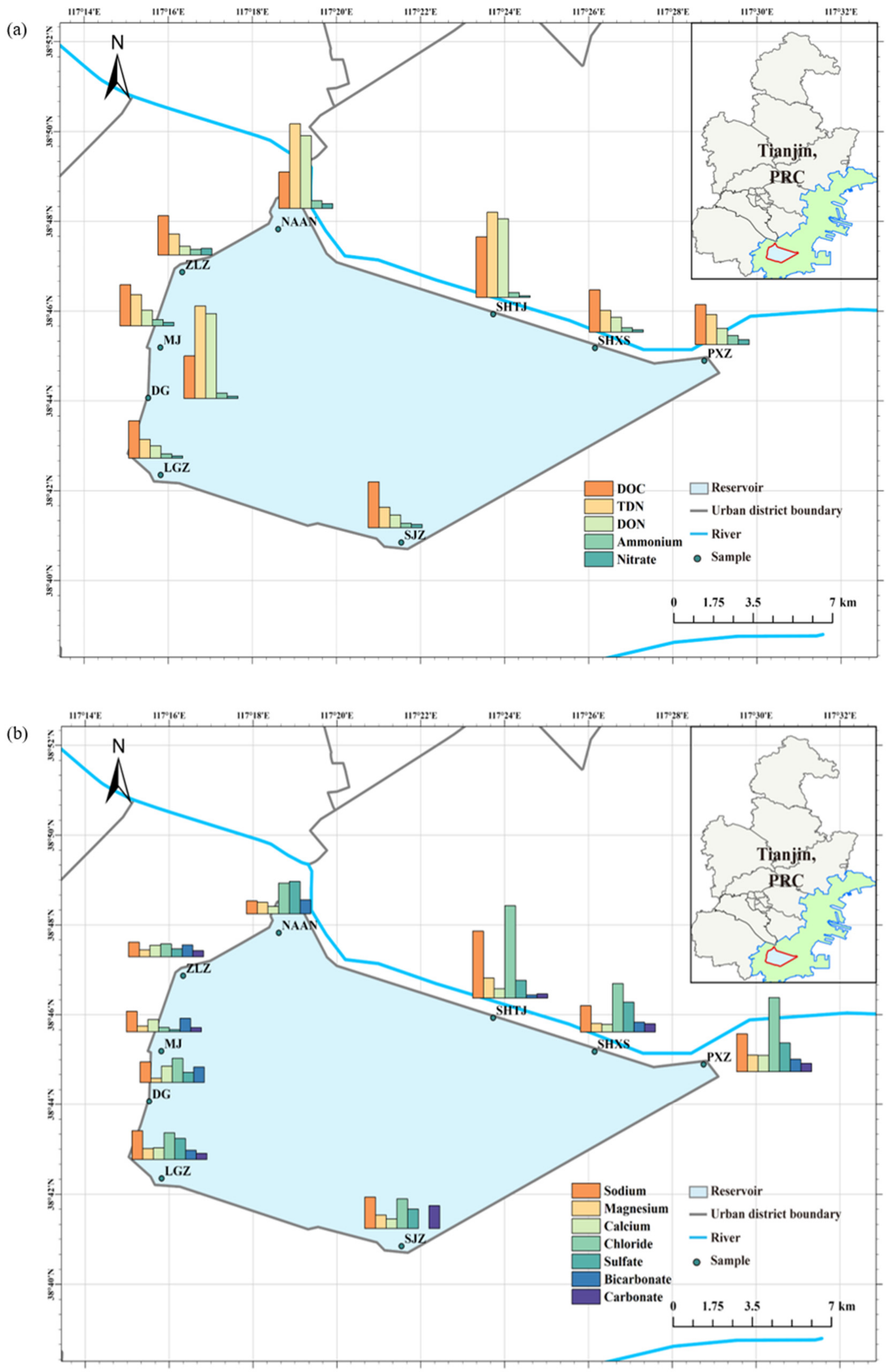

Figure S2. Concentrations of organic matters and inorganic ions. (a) Concentrations of organic constitutions (mg L^{-1}) in nine water samples. (b) Concentrations of inorganic species (meq L^{-1}) in the nine samples.

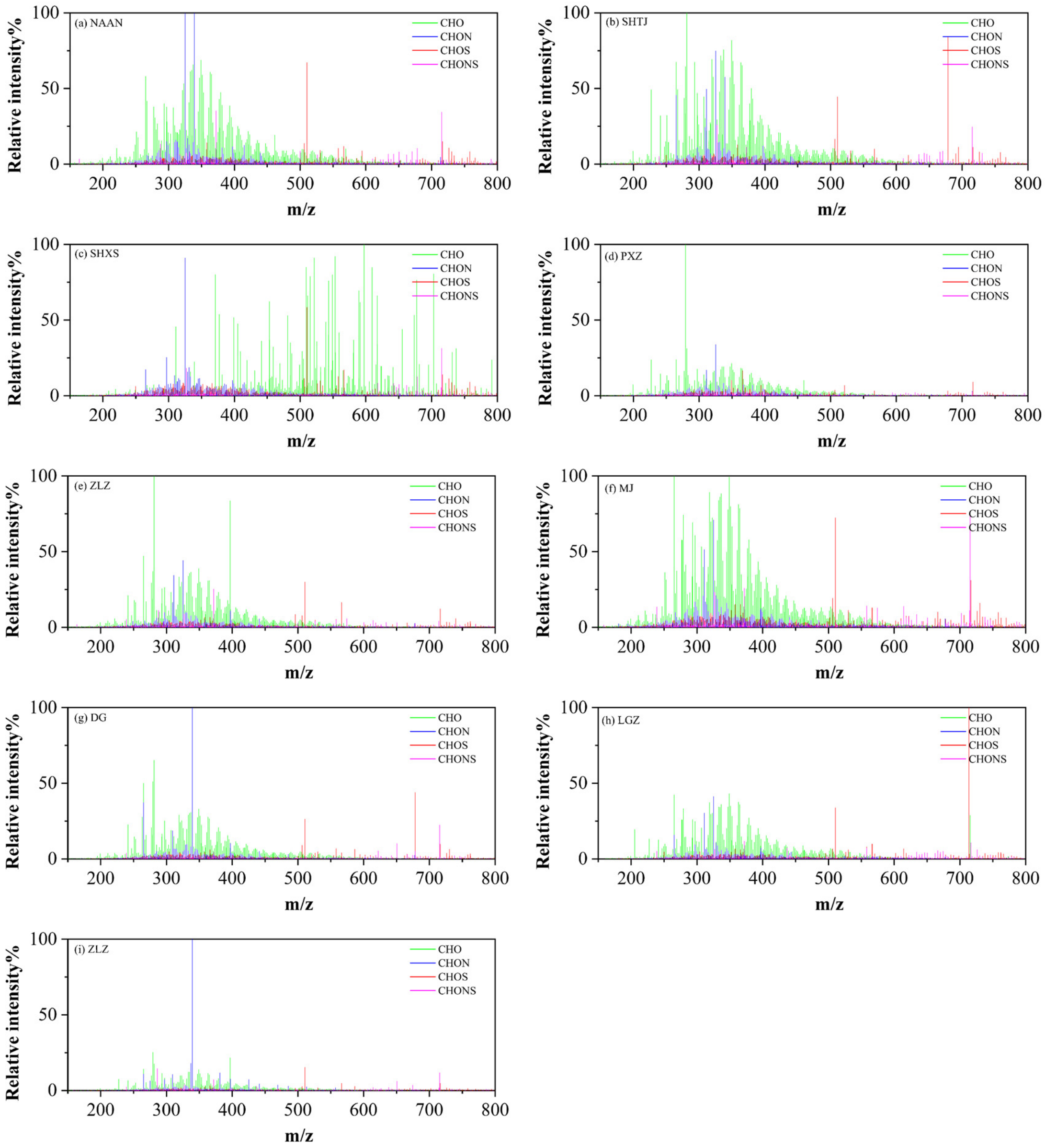

Figure S3. Reconstructed Fourier-transform ion cyclotron resonance mass spectra for the nine water samples. Signal intensities were normalized separately for each sample, with the highest peak set to 100%.

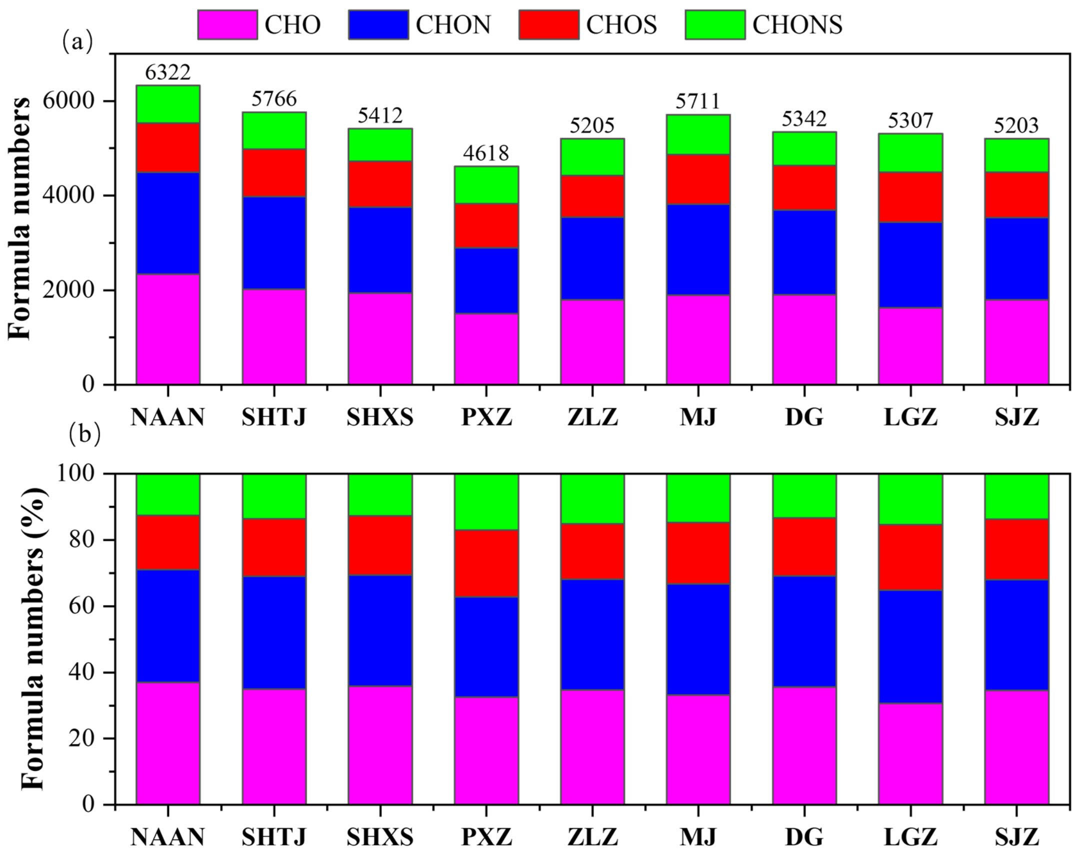

Figure S4. Statistical distribution of CHO, CHON, CHOS, and CHONS formulas across water samples. (a) Stacked bar charts show the number of assigned formulas in each class. Numbers above the bars indicate the total number of assigned formulas. (b) Relative contributions (%) of each class to the total assigned formulas.

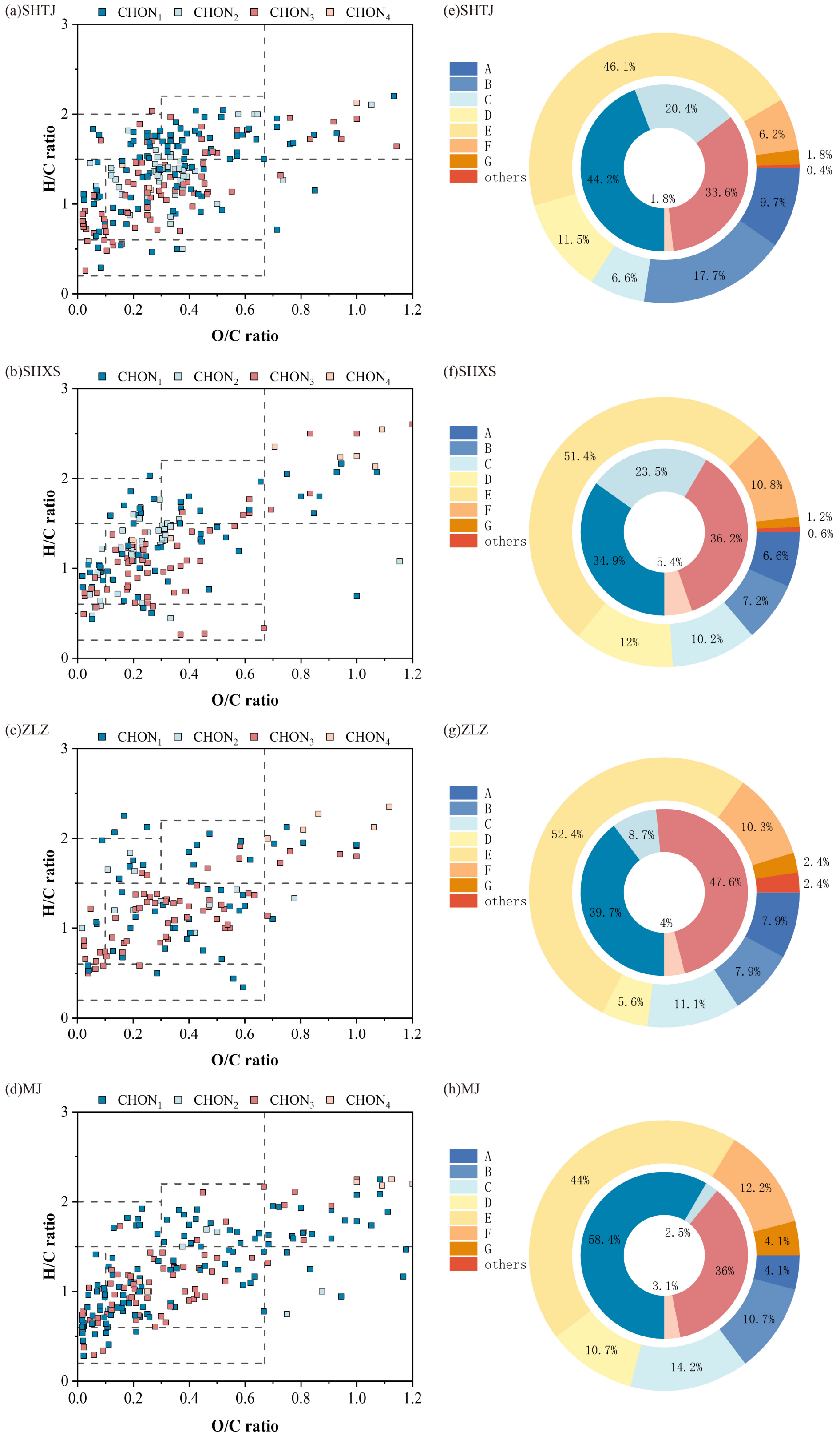

Figure S5. Van Krevelen diagrams (a-d), relative abundances and Bianco subgroups (e-h) of sample-specific CHON formulas in SHTJ, SHXS, ZLZ, MJ.

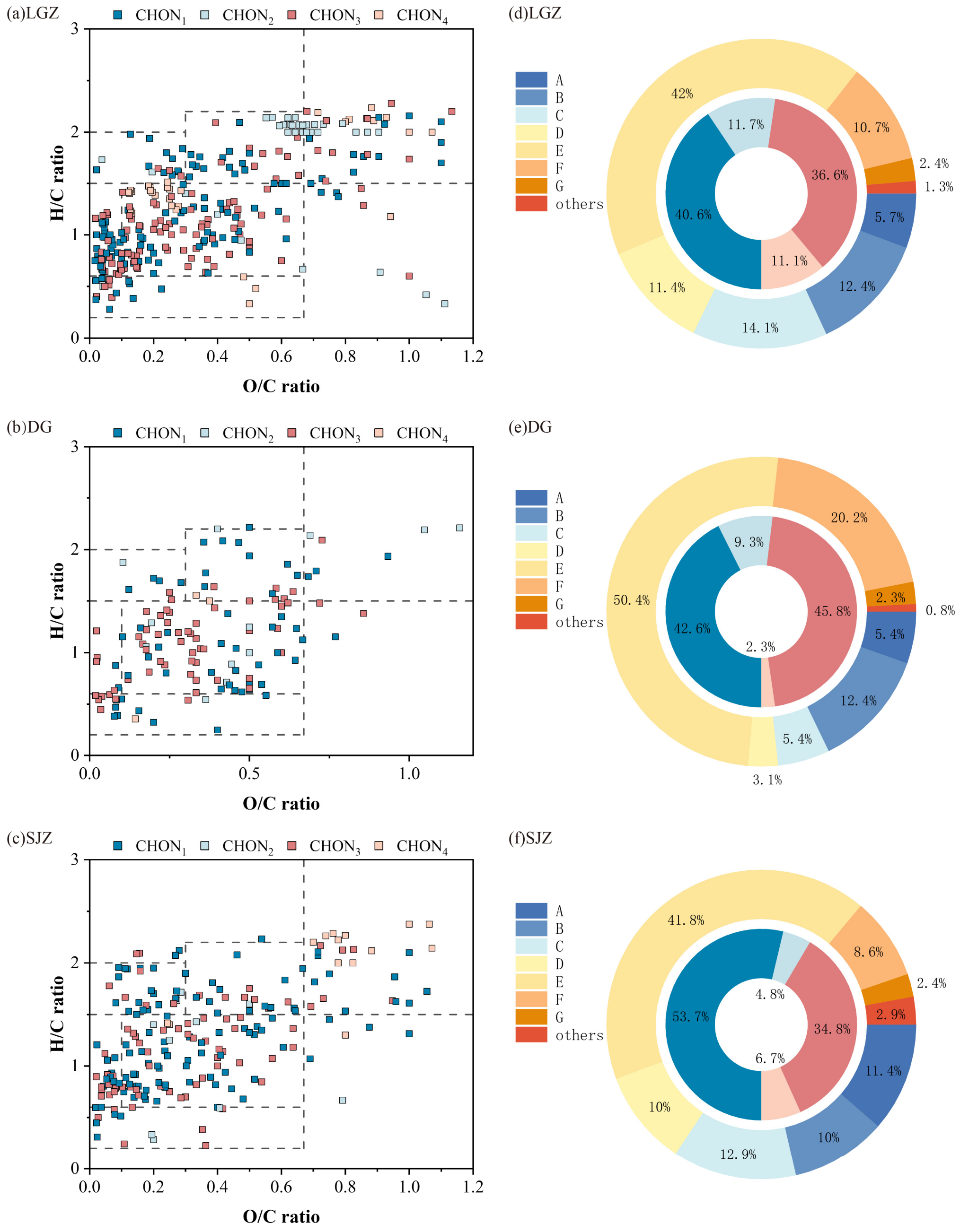

Figure S6. Van Krevelen diagrams (a-c), relative abundances and Bianco subgroups (d-f) of sample-specific CHON formulas in LGZ, DG, SJZ.

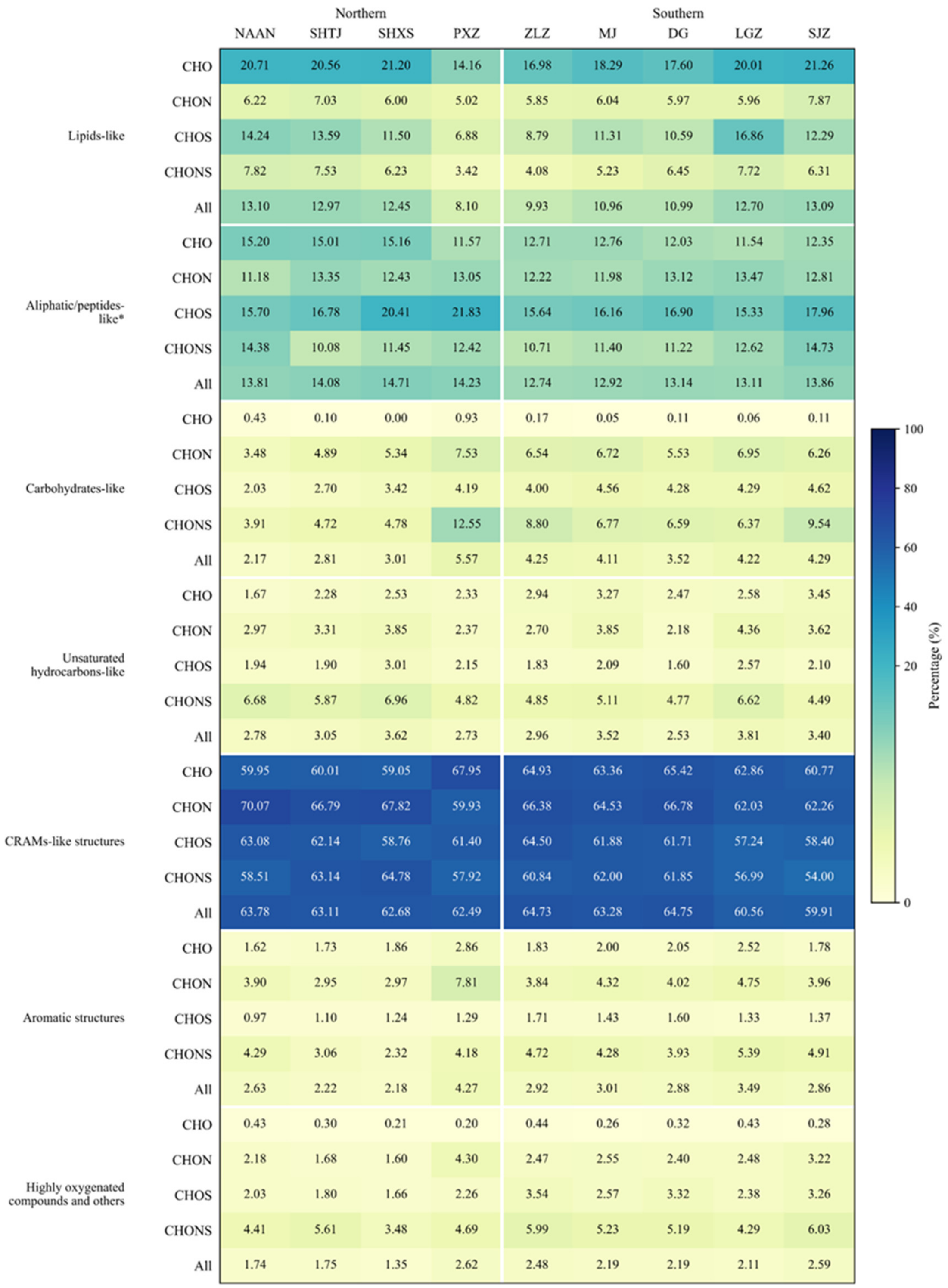

*: Nitrogen-free formulas (CHO and CHOS) were classified as aliphatic-like, whereas nitrogen-containing formulas (CHON and CHONS) were classified as peptide-like.

Figure S7. Heatmap of molecular subgroup percentages across sampling sites in the Beidagang Reservoir. Percentages are calculated within each elemental group (CHO, CHON, CHOS, and CHONS), whereas "All" represents the total of all elemental groups in each sample. Northern and southern sampling groups are separated by a vertical white line.

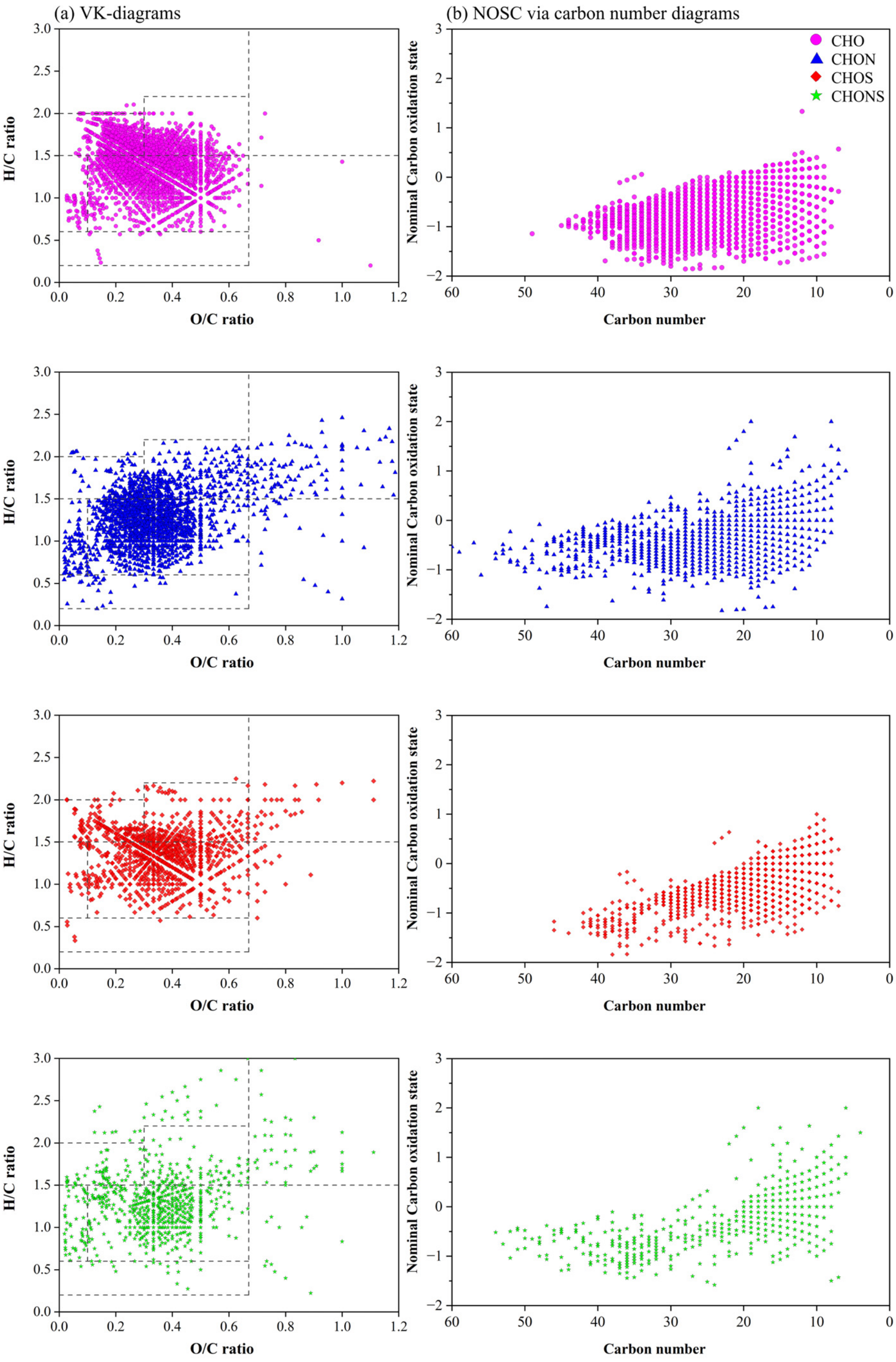

Figure S8. Van Krevelen diagrams (a) and nominal carbon oxidation state (NOSC) via carbon number (b) of assigned formulas in SHTJ. CHO, CHON, CHOS, and CHONS formulas are shown separately from top to bottom.

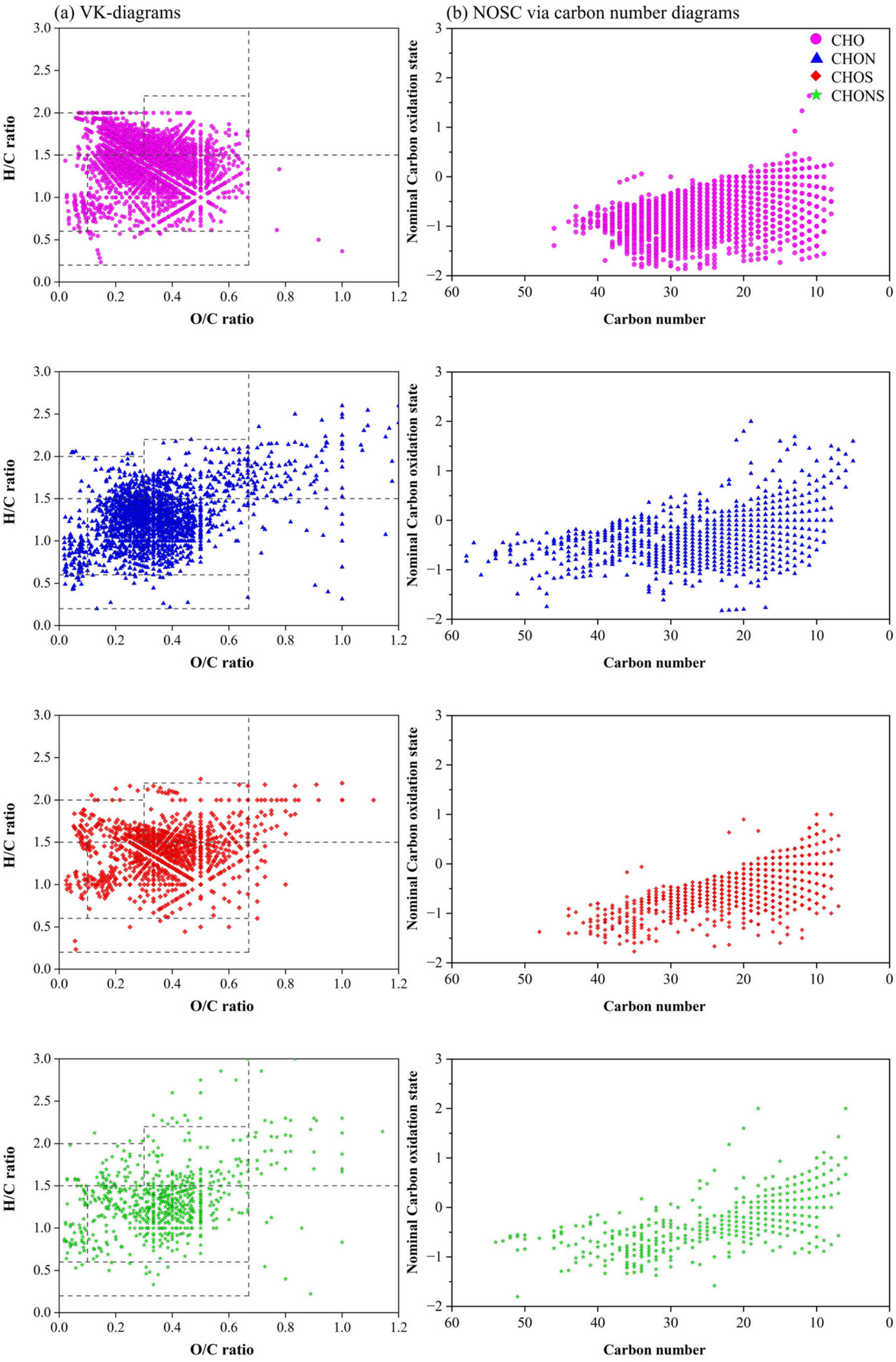

Figure S9. Van Krevelen diagrams (a) and nominal carbon oxidation state (NOSC) via carbon number (b) of assigned formulas in SHXS. CHO, CHON, CHOS, and CHONS formulas are shown separately from top to bottom.

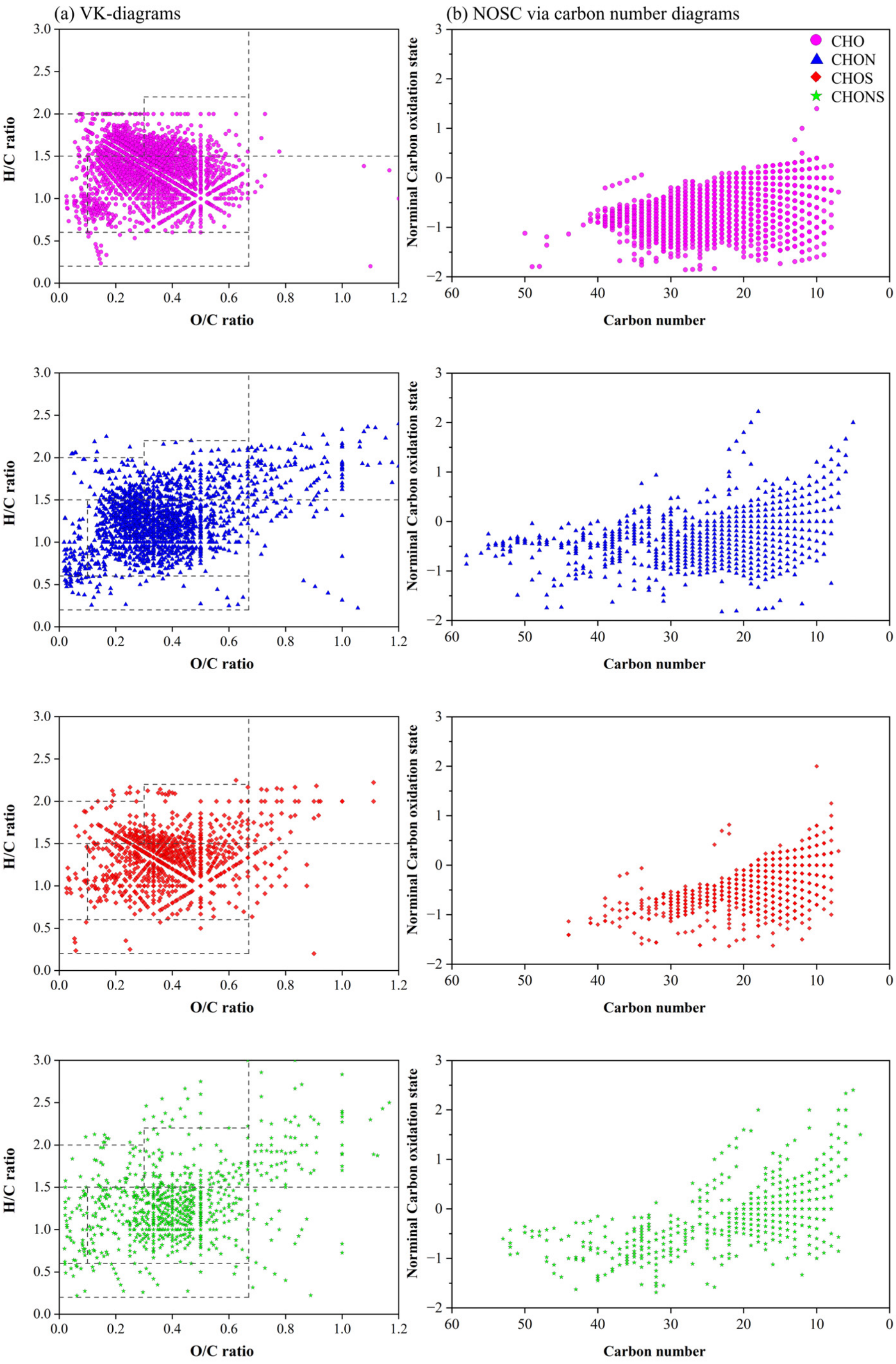

Figure S10. Van Krevelen diagrams (a) and nominal carbon oxidation state (NOSC) via carbon number (b) of assigned formulas in ZLZ. CHO, CHON, CHOS, and CHONS formulas are shown separately from top to bottom.

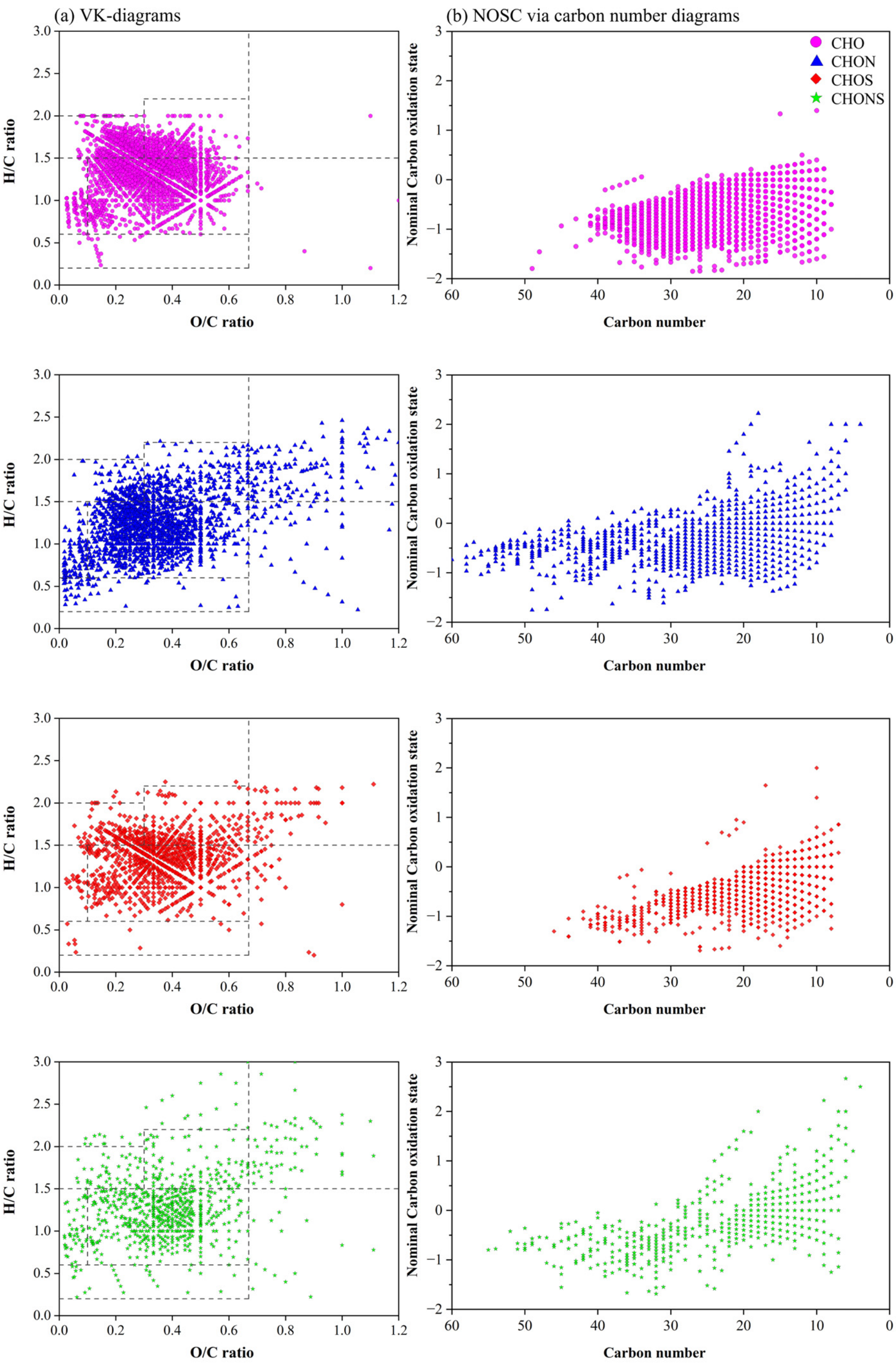

Figure S11. Van Krevelen diagrams (a) and nominal carbon oxidation state (NOSC) via carbon number (b) of assigned formulas in MJ. CHO, CHON, CHOS, and CHONS formulas are shown separately from top to bottom.

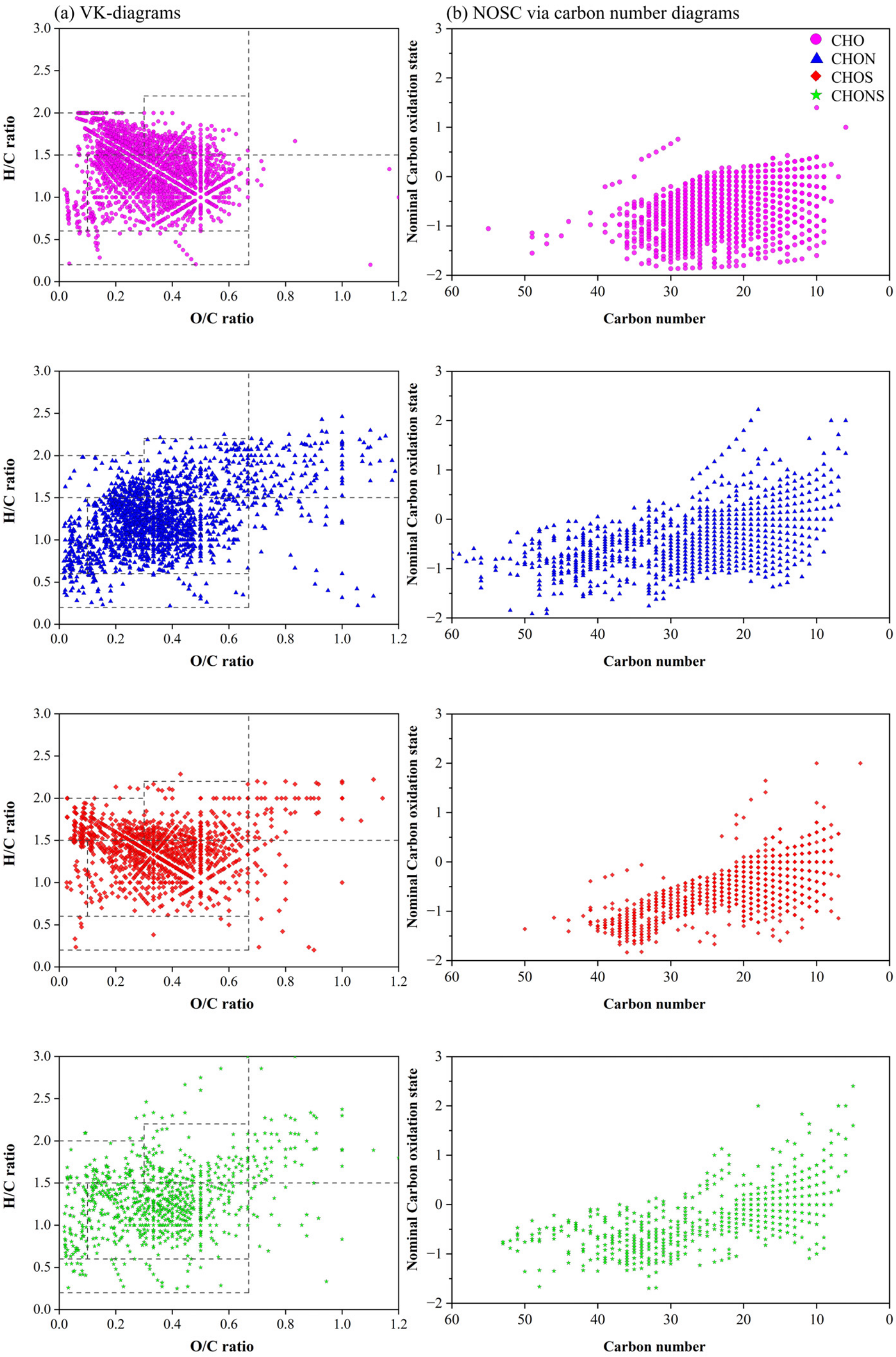

Figure S12. Van Krevelen diagrams (a) and nominal carbon oxidation state (NOSC) via carbon number (b) of assigned formulas in LGZ. CHO, CHON, CHOS, and CHONS formulas are shown separately from top to bottom.

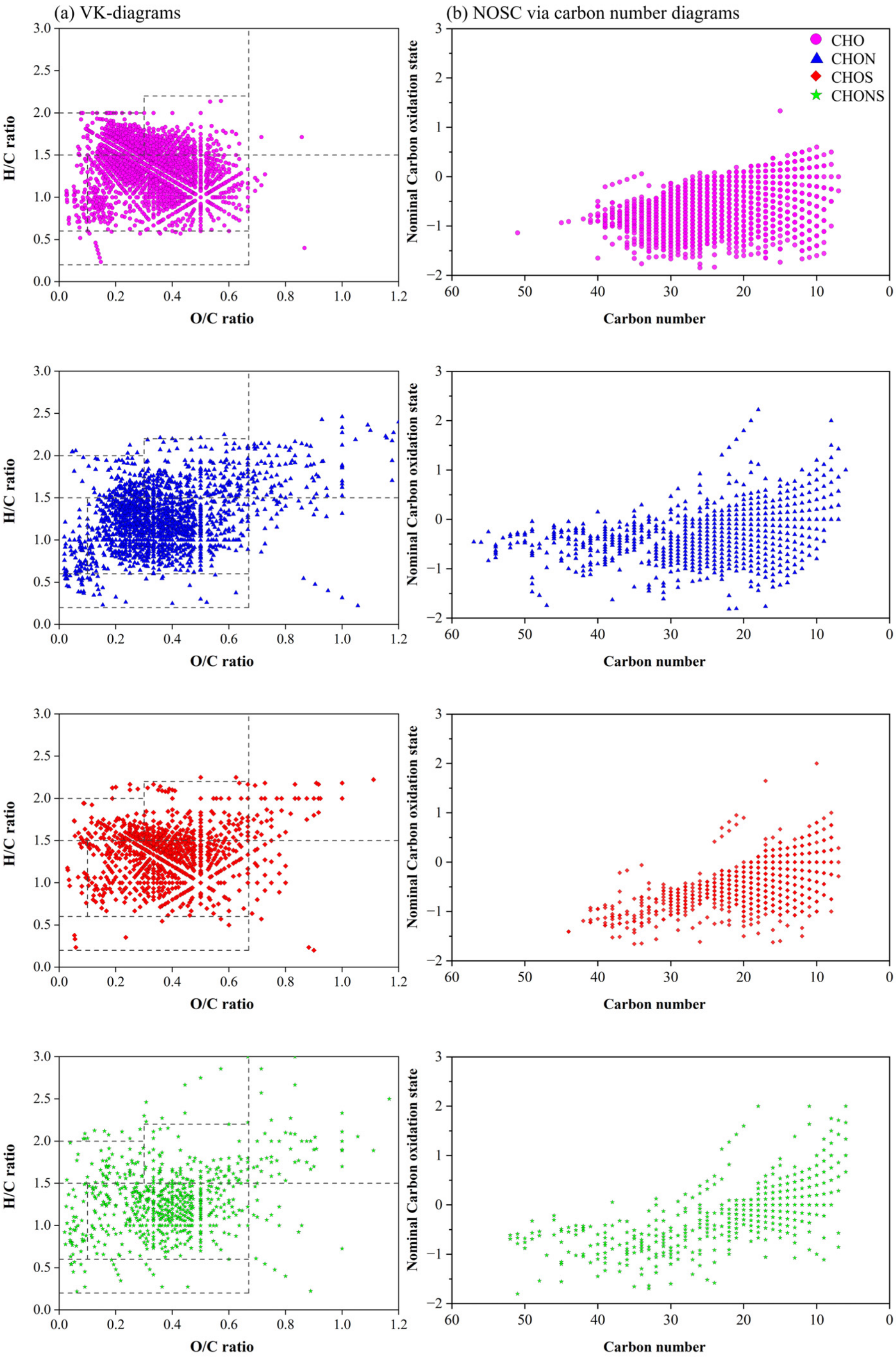

Figure S13. Van Krevelen diagrams (a) and nominal carbon oxidation state (NOSC) via carbon number (b) of assigned formulas in DG. CHO, CHON, CHOS, and CHONS formulas are shown separately from top to bottom.

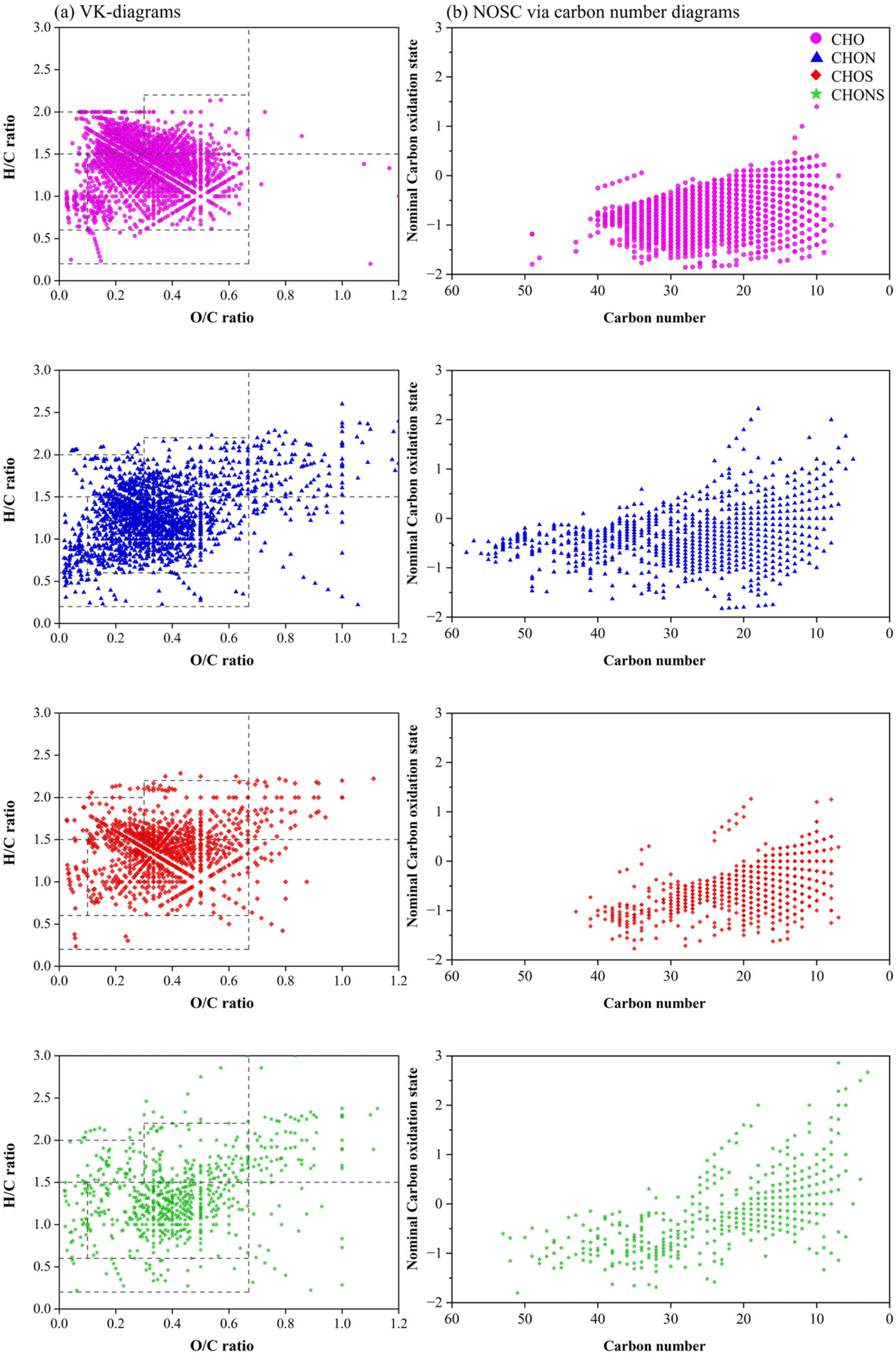

Figure S14. Van Krevelen diagrams (a) and nominal carbon oxidation state (NOSC) via carbon number (b) of assigned formulas in SJZ. CHO, CHON, CHOS, and CHONS formulas are shown separately from top to bottom.

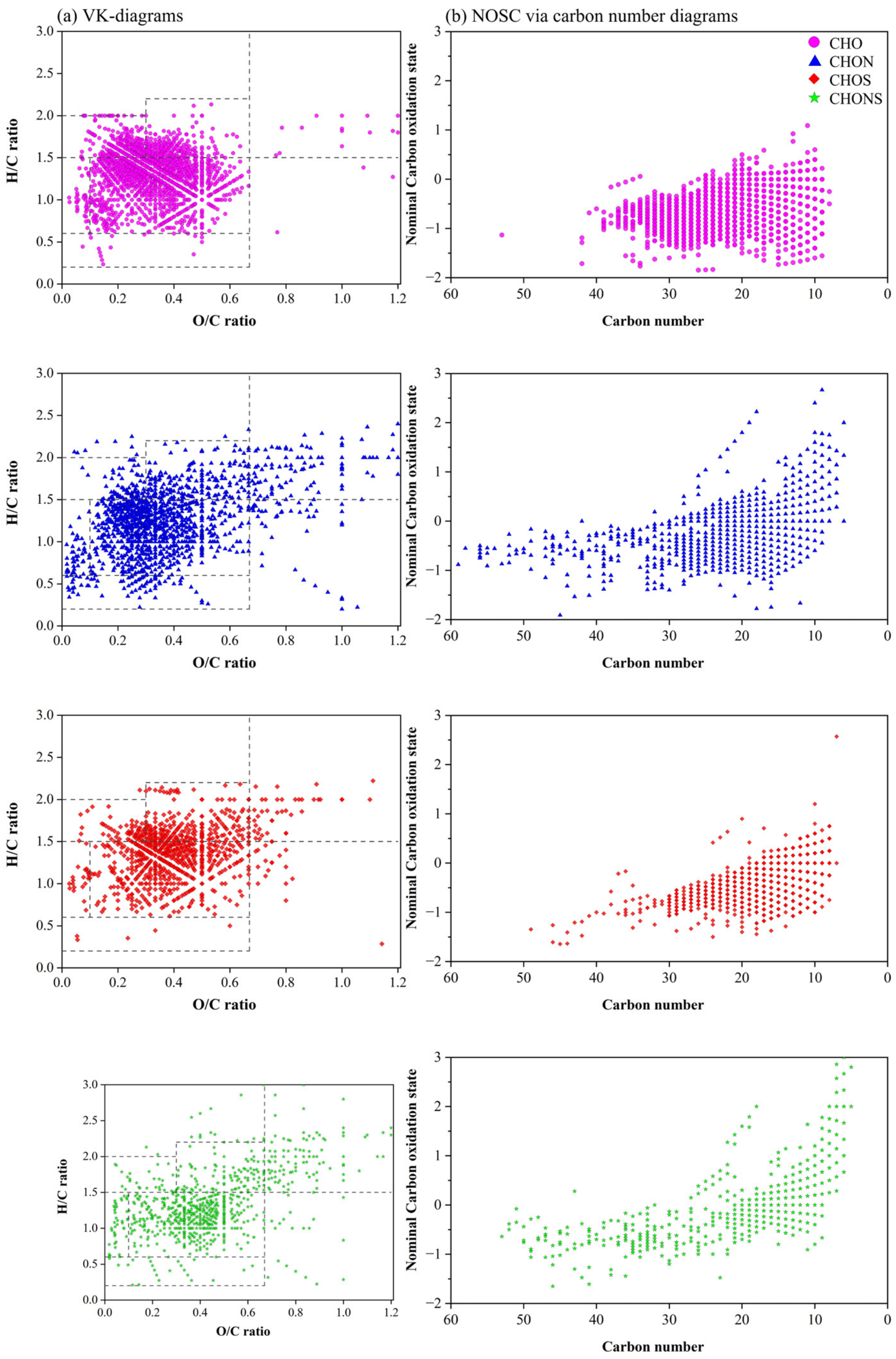

Figure S15. Van Krevelen diagrams (a) and nominal carbon oxidation state (NOSC) via carbon number (b) of assigned formulas in PXZ. CHO, CHON, CHOS, and CHONS formulas are shown separately from top to bottom.

References

1. LaRowe, D.E.; Van Cappellen, P. Degradation of natural organic matter: A thermodynamic analysis. *Geochimica et Cosmochimica Acta* **2011**, *75*, 2030-2042.
2. Koch, B.P.; Witt, M.; Engbrodt, R.; Dittmar, T.; Kattner, G. Molecular formulae of marine and terrigenous dissolved organic matter detected by electrospray ionization fourier transform ion cyclotron resonance mass spectrometry. *Geochimica et Cosmochimica Acta* **2005**, *69*, 3299-3308.
3. Bianco, A.; Deguillaume, L.; Vaitilingom, M.; Nicol, E.; Baray, J.L.; Chaumerliac, N.; Bridoux, M. Molecular characterization of cloud water samples collected at the puy de dome (france) by fourier transform ion cyclotron resonance mass spectrometry. *Environmental Science & Technology* **2018**, *52*, 10275-10285.
4. Rodriguez-Cardona, B.M.; Coble, A.A.; Wymore, A.S.; Kolosov, R.; Podgorski, D.C.; Zito, P.; Spencer, R.G.M.; Prokushkin, A.S.; McDowell, W.H. Wildfires lead to decreased carbon and increased nitrogen concentrations in upland arctic streams. *Scientific Reports* **2020**, *10*, 8722.
5. LaBrie, R.; Pequin, B.; Fortin St-Gelais, N.; Yashayaev, I.; Cherrier, J.; Gelinas, Y.; Guillemette, F.; Podgorski, D.C.; Spencer, R.G.M.; Tremblay, L.; Maranger, R. Deep ocean microbial communities produce more stable dissolved organic matter through the succession of rare prokaryotes. *Sci Adv* **2022**, *8*, eabn0035.
6. Kim, M.S.; Wang, Y.; Choi, M.; Chen, S.; Bae, M.-S.; Park, K.; Hu, M.; Jang, K.-S. Comparison of secondary organic aerosol (soa)-associated molecular features at urban sites in china and korea in winter and summer (2019). *Atmospheric Environment* **2024**, *318*,
7. Hemingway, J.D.; Spencer, R.G.M.; Podgorski, D.C.; Zito, P.; Sen, I.S.; Galy, V.V. Glacier meltwater and monsoon precipitation drive upper ganges basin dissolved organic matter composition. *Geochimica et Cosmochimica Acta* **2019**, *244*, 216-228.
8. He, C.; Yi, Y.; He, D.; Cai, R.; Chen, C.; Shi, Q. Molecular composition of dissolved organic matter across diverse ecosystems: Preliminary implications for biogeochemical cycling. *Journal of Environmental Management* **2023**, *344*, 118559.
9. Guo, D.; Zhang, R.; Ma, H.; Zhang, H.; Zhang, Z.; Qi, Y.; Sun, P.; He, D.; Li, S.L. Photochemical transformation of dissolved organic matter: Source, particulate matter interaction and disinfection byproduct formation potential. *Water Research* **2026**, *290*, 125078.
10. Chen, S.; Xie, Q.; Su, S.; Wu, L.; Zhong, S.; Zhang, Z.; Ma, C.; Qi, Y.; Hu, W.; Deng, J.; Ren, L.; Zhu, D.; Guo, Q.; Liu, C.Q.; Jang, K.S.; Fu, P. Source and formation process impact the chemodiversity of rainwater dissolved organic matter along the yangtze river basin in summer. *Water Research* **2022**, *211*, 118024.
11. Zhao, G.; Wang, L.; Li, Y.; Chen, J.; Niu, L.; Lin, L. Machine learning uncovers tidal dom transformations and keystone molecules via ft-icr ms and reactomics for estuarine nutrient cycling. *Journal of Environmental Sciences* **2026**, *166*, 570-580.
12. Hou, C.; Chen, L.; Dong, Y.; Yang, Y.; Zhang, X. Unraveling dissolved organic matter in drinking water through integrated ozonation/ceramic membrane and biological activated carbon process using ft-icr ms. *Water Research* **2022**, *222*, 118881.
13. Su, S.; Xie, Q.; Lang, Y.; Cao, D.; Xu, Y.; Chen, J.; Chen, S.; Hu, W.; Qi, Y.; Pan, X.; Sun, Y.; Wang, Z.; Liu, C.Q.; Jiang, G.; Fu, P. High molecular diversity of organic nitrogen in urban snow in north china. *Environmental Science & Technology* **2021**, *55*, 4344-4356.

14. Zhao, C.; Zhang, H.; Li, P.; Yi, Y.; Zhou, Y.; Wang, Y.; He, C.; Shi, Q.; He, D. Dissolved organic matter cycling revealed from the molecular level in three coastal bays of china. *Science of the Total Environment* **2023**, *904*, 166843.
